# Fatty Acid Oxidation‐Glycolysis Metabolic Transition Affects ECM Homeostasis in Silica‐Induced Pulmonary Fibrosis

**DOI:** 10.1002/advs.202407134

**Published:** 2024-12-25

**Authors:** Wenqing Sun, Siyun Zhou, Lan Peng, Wei Wang, Yi Liu, Ting Wang, Demin Cheng, Ziwei Li, Haojie Xiong, Xinying Jia, Wenxiu Lian, Jiandong Jiao, Chunhui Ni

**Affiliations:** ^1^ The Affiliated Wuxi Center for Disease Control and Prevention of Nanjing Medical University Wuxi Center for Disease Control and Prevention Wuxi Medical Center Nanjing medical university Nanjing 211166 China; ^2^ Department of Occupational Medical and Environmental Health Key Laboratory of Modern Toxicology of Ministry of Education Center for Global Health School of Public Health Nanjing Medical University Nanjing 211166 China; ^3^ Department of Pathology Nanjing Drum Tower Hospital The Affiliated Hospital of Nanjing University Medical School Nanjing 210000 China; ^4^ Department of Public Health Kangda College of Nanjing Medical University Lianyungang 320700 China

**Keywords:** extracellular matrix, fatty acid oxidation, glycolysis, hypoxia‐inducible factor‐1α, silicosis

## Abstract

Silicosis is a fatal occupational pulmonary disease that is characterized by irreversible replacement of lung parenchyma by aberrant Exracellular matrix (ECM). Metabolic reprogramming is a crucial mechanism for fibrosis. However, how the metabolic rewiring shifts the ECM homeostasis toward overaccumulation remains unclear. Herein, a phenotype with reduction in fatty acid oxidation (FAO) but enhanced glycolysis in myofibroblasts is shown. Perturbation of the glycolytic and FAO pathways, respectively, reveals distinct roles in the metabolic distribution of ECM deposition and degradation. Suppressed glycolysis leads to a decrease in insoluble ECM, primarily due to the inhibition of ECM‐modifying enzyme activity and a decrease in glycine synthesis. Notably, promoted FAO facilitates the intracellular degradation pathway of ECM. In addition, the findings revealed that hypoxia‐inducible factor‐1 alpha (HIF‐1α) serves as a crucial metabolic regulator in the transition from FAO to glycolysis, thereby playing a significant role in ECM deposition in silica‐induced pulmonary fibrosis. Further, the promotion of FAO, inhibition of glycolysis and HIF‐1α reduce ECM production and promote ECM degradation, ultimately impeding the progression of fibrosis and providing therapeutic relief for established pulmonary fibrosis in vivo. These findings unveil the metabolic rewire underpinning the deposition of ECM in silica‐induced lung fibrosis and identify novel targets for promoting regression of pulmonary fibrosis.

## Graphical abstract

Increased glycolysis and decreased FAO contribute to the development of silica dust‐induced pulmonary fibrosis via promoting the dysregulation of ECM synthesis and degradation. HIF‐1α regulates ECM remodeling via regulating the FAO‐glycolysis metabolic shift. The promotion of FAO, inhibition of glycolysis and HIF‐1α reduce ECM production and promote ECM degradation, ultimately impeding the progression of fibrosis and providing therapeutic relief for established pulmonary fibrosis.

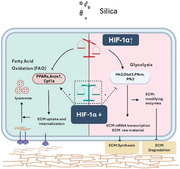

## 1 Introduction

Pulmonary fibrosis is a chronic and fatal interstitial lung disease initiated by dysregulated wound healing in response to injury, resulting in pulmonary dysfunction and even death. It represents one end of a spectrum of lung responses to injury caused by infectious, environmental exposures, infectious, occupational exposures, toxic, autoimmunity, or other risk factors. To date, pulmonary fibrosis remains an unmet medical need. Long‐term exposure to inhalable silica dust in the occupational environment can cause a pulmonary disease termed silicosis, characterized by diffuse fibrosis of the lungs. Silicosis is the most common, fastest progressing, and most serious occupational disease among pneumoconiosis.^[^ ^1^ ^]^ Therefore, pneumoconiosis (especially silicosis) will still bring a heavy economic burden to individuals and society in the future for a long time, which is a severe public health problem that cannot be ignored. The complicated pathogenesis of silicosis limits the progress of drug development. Exploring the pathological processes and identifying the key regulatory factors and specific biomarkers, which have vital value for the early diagnosis, intervention, and development of specific drugs.

Fibrosis is mainly characterized by abnormal proliferation of myofibroblasts and excessive production of ECM, resulting in the disruption of the typical lung architecture and functional impairment. The ECM is a dynamically regulated framework present in all tissues, undergoing continual controlled restructuring to uphold standard tissue and cellular operations.^[^ ^2^ ^]^ In fibrotic conditions, alterations in the composition and physicochemical characteristics of the ECM suggest that certain ECM components could serve as promising diagnostic indicators and therapeutic targets.^[^ ^3^ ^]^ Recent studies have found that fibrotic ECM is not solely a consequence of the disease, but also functions as a central profibrotic factor.^[^ ^4^ ^]^ The fibrotic ECM transmits pro‐fibrotic biochemical and biomechanical signals that stimulate inflammation and persistent fibroblast activation, perpetuating a continuous pro‐fibrotic feedback loop.^[^ ^5^ ^]^ Consequently, removal of the initial fibrogenic insult does not lead to reversal of fibrosis. Therefore, it is imperative to consider the removal of fibrotic ECM in the formulation of anti‐fibrosis strategies and the development of efficacious therapeutic drugs. This is essential not only for the restoration of the organ's normal physiological structure but also for impeding the advancement of progressive fibrosis.

In pulmonary fibrosis, metabolic reprogramming or dysregulation occurs in multiple cells, such as fibroblasts, macrophages, epithelial cells, and other cells, and promotes fibrosis processing by affecting autophagy, cell proliferation, cell apoptosis, inflammatory response, synthesis, and secretion of ECM and other pathological mechanisms.^[^ ^6^ ^]^ Although glycolysis produces less adenosine triphosphate (ATP) than oxidative phosphorylation (OXPHOS), glycolysis reacts faster and provides energy and raw materials for the proliferation and ECM secretion of myoblasts in a short period. Studies have confirmed that the increase of glycolysis in fibrosis is accompanied by a decrease in the level of FAO.^[^ ^7^ ^]^ Damage to FAO leads to the accumulation of fatty acids, which are poor biosynthetic precursors. Whether glycolytic‐FAO metabolic disturbance transforms ECM homeostasis into ECM over‐deposition in lung fibroblasts remains to be further investigated.

Glucose plays a crucial role as a primary provider of cellular energy and a fundamental precursor for the biosynthesis of nucleotides, lipids, and amino acids. In the process of glycolysis, every glucose molecule generates 2 molecules of ATP. Subsequently, the resulting pyruvate undergoes further metabolic processes within the mitochondria, leading to the production of either lactate or acetyl‐coenzyme A. Although glycolysis conventionally occurs in anaerobic conditions, recent studies have demonstrated its occurrence in aerobic environments as well. The phenomenon of aerobic glycolysis, also referred to as the “Warburg effect”, was initially identified in tumor research and has since become a focal point of interest. Substantial evidence indicates that aerobic glycolysis is prevalent in numerous fibrotic conditions, including liver, kidney, heart, lung, and skin fibrosis.^[^ ^8^ ^]^ Our previous study further demonstrated increased glycolytic activity in lung tissues of mice with silica dust‐induced pulmonary fibrosis and in transforming growth factor‐beta 1 (TGF‐β1)‐induced activated fibroblasts.^[^ ^9^ ^]^ Like the Warburg effect, glycolysis is essential for providing energy and essential components for ECM synthesis. Specifically, the intermediate metabolite of glycolysis, 3‐phosphoglycerate (3‐PG), undergoes a catalytic conversion to produce glycine, a crucial amino acid for collagen synthesis, which accounts for one‐third of its amino acid composition.^[^ ^10^ ^]^ Additionally, the production of lactate through glycolysis has been demonstrated to enhance the activity of proline hydroxylase, thereby promoting collagen hydroxylation.^[^ ^11^ ^]^ Further investigation is necessary to comprehensively elucidate the exact molecular mechanisms underlying the relationship between glycolysis and the ECM.

Fatty acid oxidation occurs in mitochondria or peroxisomes, producing ATP as a crucial cellular energy source. In renal fibrosis, heightened fatty acid oxidation has been demonstrated to attenuate fibrotic markers, alleviate the inflammatory response, protect epithelial cells, and reduce macrophage infiltration.^[^ ^12^ ^]^ Conversely, in dermal fibrosis, TGF‐β1 inhibits fatty acid oxidation in dermal fibroblasts, leading to excessive extracellular matrix synthesis, whereas the inhibition of glycolysis has an opposing effect.^[^ ^13^ ^]^ Previous study has demonstrated that TGF‐β1 reduces fatty acid oxidation and increases fatty acid synthase (FASN) expression in lung fibroblasts.^[^ ^14^ ^]^ In idiopathic pulmonary fibrosis (IPF), there is an increase in glycolysis and a decrease in fatty acid oxidation in lung fibroblasts.^[^ ^7b^ ^]^ Perturbing these metabolic pathways can influence fibroblast differentiation. Therefore, modulating the equilibrium between glycolysis and fatty acid oxidation in fibroblasts presents a potentially effective strategy for augmenting extracellular matrix breakdown and mitigating fibrosis.

HIF‐1α can regulate the expression of several hypoxia‐regulating genes to regulate various critical biological pathways such as cell proliferation, energy metabolism, invasion, and metastasis. HIF‐1α is not only a vital regulatory factor of glycolysis but also regulates other metabolic processes, such as fatty acid metabolism and amino acid metabolism.^[^ ^15^ ^]^ However, it is unclear whether HIF‐1α promotes ECM deposition in silica‐induced pulmonary fibrosis by regulating the metabolic transition of FAO‐glycolysis.

In this study, we show a metabolic perturbation of glycolysis and FAO in fibroblast activation for excessive ECM deposition and investigate the consequences of this metabolic shift in ECM synthesis, modification, and degradation. In addition, we reveal the functions of HIF‐1α in regulating ECM deposition through the modulation of glycolytic‐FAO metabolic pathways. This study seeks to evaluate the therapeutic potential of targeting glycolysis, FAO, and HIF‐1α in silica‐induced pulmonary fibrosis, thus highlighting the potential of HIF‐1α‐mediated metabolic dysregulation as a promising therapeutic target for experimental pulmonary fibrosis.

## 2 Results

### 2.1 The Development of Pulmonary Fibrosis in Mice is Accompanied by a Restructuring of Cellular Metabolism

**Figure 1:**
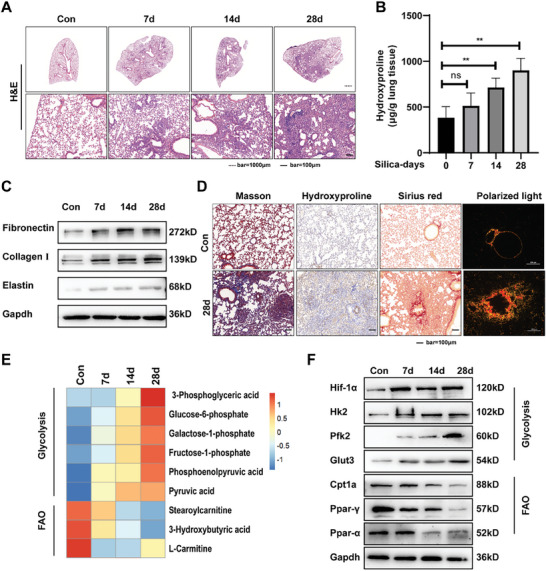
The development of pulmonary fibrosis in mice is accompanied by a restructuring of cellular metabolism. A) Pathological changes in mouse lung tissues were presented by H&E staining (*n* = 6 for each group); dashed line scale bar = 1000 µm, solid line scale bar = 100 µm. B)Hydroxyproline content of the lung tissues was used to examine the degree of collagen deposition. All data were expressed as the means ± SD of at least 3 independent experiments, ^*^ *p* < 0.05 and ^**^ *p* < 0.01.C) The protein levels of fibronectin, collagen I, and elastin in each group were examined by the western blot. The results of the experiment were repeated at least 3 times. D) Histological analysis was conducted on mouse lung tissue using Masson staining (first image from left), Hydroxyproline immunohistochemical staining (second image from left), Sirius red staining (second image from right), and polarized light imaging (first image from right); scale bar = 100 µm. E) The heatmap of differential metabolites about glycolysis and FAO identified by metabolomics.F) Protein levels of key molecules involved in glycolysis and fatty acid oxidation in TGF‐β1‐treated MRC‐5 cells were quantified using western blot analysis. Each experiment was performed in triplicate to ensure reproducibility of results.

To analyze cell metabolic state in response to silica stimulation, we utilized a single intratracheal instillation of silica suspension mouse model and collected lung tissues from mice at different time points (7day, 14day, 28day) after silica exposure. As shown in the results of H&E staining (**Figure** **1**A), as silicosis progressed, lung inflammation and fibrosis continuously got worse. The hydroxyproline assay revealed a progressive elevation in collagen levels within lung tissue following extended exposure to silica dust (Figure 1B). The expression of ECM components (fibronectin, collagen I, elastin) also increased during silicosis progression (Figure 1C; Figure S1A, Supporting Information). To gain further insight into the deposition of collagen in silica‐induced mouse fibrotic lung tissue, the pathological sections of mouse lung tissue were stained to determine the content of collagen in different forms. The results of Masson staining, hydroxyproline immunohistochemical staining, collagen I immunofluorescence staining and Sirius red staining showed that fibrotic mouse lung collagen levels remained steadily high (Figure 1D; Figure S1B, Supporting Information).

Using lung tissues from silica‐exposure mice, we conducted analysis of transcriptomic and metabolomics to characterize the metabolic state. Transcriptomic findings indicated that fibrosis marker genes were significantly up‐regulated, and ECM as well as collagen‐related ECM genes were markedly increased. Additionally, pathway enrichment analysis revealed that ECM deposition augmented 14 days after injury and was accompanied by alterations in glycolipid metabolism (Figure S1C–E, Supporting Information). More importantly, the transcript expression of the key characteristic glycolytic genes was examined in the RNAseq data, which revealed that genes like Hif‐1α, phosphofructokinase‐1 (Pfk2), hexokinase 2 (Hk2), and lactate dehydrogenase A (Ldha) were upregulated in mouse lung tissues with silica‐induced fibrosis compared to control. Conversely, a downregulation of catalytic rate‐limiting factors for fatty acid oxidation was observed (Figure S1C, Supporting Information). Analysis of metabolites using metabolomics demonstrated an increase in certain glycolytic intermediates, while products of fatty acid oxidation were decreased (Figure 1E). Furthermore, the protein levels of glycolytic regulators (Hif‐1α, Hk2, glucose transporter 3 (Glut3), Pfk2) exhibited an increase, and conversely, regulators related to fatty acid oxidation were decreased, as assessed by Western blot (Figure 1F; Figure S1F, Supporting Information). Together, these findings uncover a metabolic shift in mouse lung tissue during silica dust‐induced pulmonary fibrosis.

### 2.2 The Deposition of ECM by Activated Fibroblasts is Accomplished by a Metabolic Perturbation of Glycolysis and FAO

**Figure 2:**
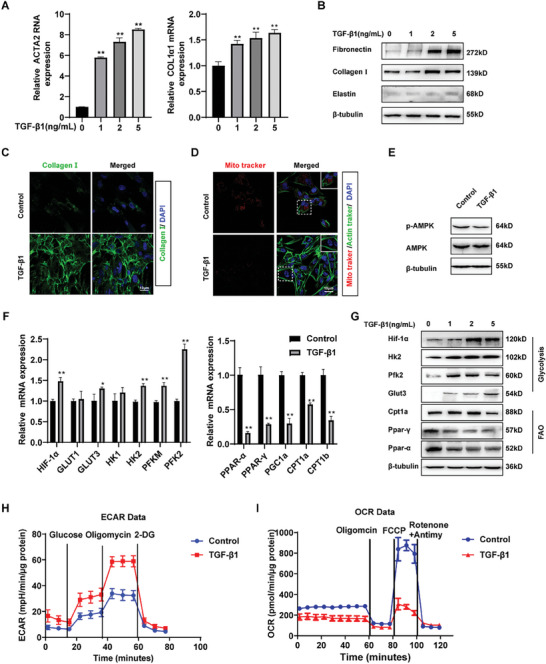
The deposition of ECM by activated fibroblasts is accomplished by a metabolic perturbation of glycolysis and FAO. A) qRT‐PCR showed that the increased in ACTA2 (the encoding gene for α‐SMA) and collagen I in MRC‐5 cells with treated with different concentrations of TGF‐β1. The data were expressed as the means ± SD of at least 3 independent experiments, ^*^ *p* < 0.05 and ^**^ *p* < 0.01. B) Western blot analysis for protein levels of core components of ECM in TGF‐β1‐treated MRC‐5 cells. The results of the experiment were repeated at least 3 times. C) Representative confocal microscopy images of immunofluorescence staining for collagen I (green) in MRC‐5 cells. Nuclei were stained with DAPI (blue). D) Mitochondrial content was measured by Mito tracker red after treatment with TGF‐β1 for 48 h, Actin tracker (green) labelling of cellular microfilaments. Nuclei were stained with DAPI (blue). E) Western blot analysis for protein levels of phosphorylated AMPK and AMPK in TGF‐β1‐treated MRC‐5 cells. The results of the experiment were repeated at least 3 times. F) Relative mRNA levels of crucial regulators of glycolysis and FAO in MRC‐5 cells treated by various concentration of TGF‐β1 by qRT‐PCR. All data were expressed as the means ± SD of at least 3 independent experiments, ^*^ *p* < 0.05 and ^**^ *p* < 0.01. G) Representative western blot images of crucial regulators of glycolysis and FAO in MRC‐5 cells treated by various concentration of TGF‐β1. The results of the experiment were repeated at least 3 times. H) Glycolysis stress test in MRC‐5 cells with or without TGF‐β1. Glucose (10 mM), oligomycin (1µM), and 2‐DG (2‐Deoxy‐Glucose, 100 mM). I) Fatty acid oxidation in MRC‐5 cells with or without TGF‐β1. Etomoxir (4 µM), oligomycin (2 µM), FCCP (1 µM), antimycin A (0.5 µM) and rotenone (0.5 µM).

Given that the synthesis and secretion of ECM is an energy‐intensive process and that myofibroblasts are the primary cell type responsible for ECM synthesis and secretion. We established a model of fibroblast activation using TGF‐β1 to test whether there is a switch in cellular metabolism during fibroblast activation. The alpha‐smooth muscle actin (α‐SMA) (encoded by the ACTA2 gene) and collagen I mRNA levels increased significantly with increasing concentrations of the TGF‐β1 treatment (**Figure** **2**A). Increased expression of ECM proteins (fibronectin, collagen I and elastin) in the activated fibroblasts, as assessed by western blot and immunofluorescence analyses (Figure 2B,C; Figure S2A, Supporting Information). Furthermore, immunofluorescence results after decellularization showed large amounts of insoluble collagen deposits (Figure S2B, Supporting Information). The labelling of mitochondria using a Mito tracker indicated a significant attenuation of mitochondrial signals after TGF‐β1 treatment (Figure 2D). In addition, there was a significant reduction in the activity of AMP‐activated protein kinase (AMPK), which is a central regulator of energy homeostasis (Figure 2E; Figure S2C, Supporting Information), indicating that metabolic alterations occur in activated fibroblasts. Using qRT‐qPCR (Figure 2F) and western blot analyses (Figure 2G; Figure S2D, Supporting Information), we validated the increased expression of key glycolytic signature genes and decreased expression of FAO‐related regulatory genes in activated fibroblasts, which is consistent with observations in lung tissue from mouse models of silicosis. Cellular metabolism was quantified by measuring extracellular acidification rates (ECAR) and oxygen consumption rates (OCR). Real‐time metabolic changes pertaining to glycolysis and glycolytic capacity were significantly increased while a lower level of FAO was observed in TGF‐β1‐treated MRC‐5 cells (Figure 2H,I). In summary, the development of lung fibrosis corresponds to the downregulation of FAO and upregulation of glycolysis in fibroblasts.

### 2.3 Inhibiting Glycolysis or Enhancing FAO affects ECM Deposition in Activated Fibroblasts

**Figure 3:**
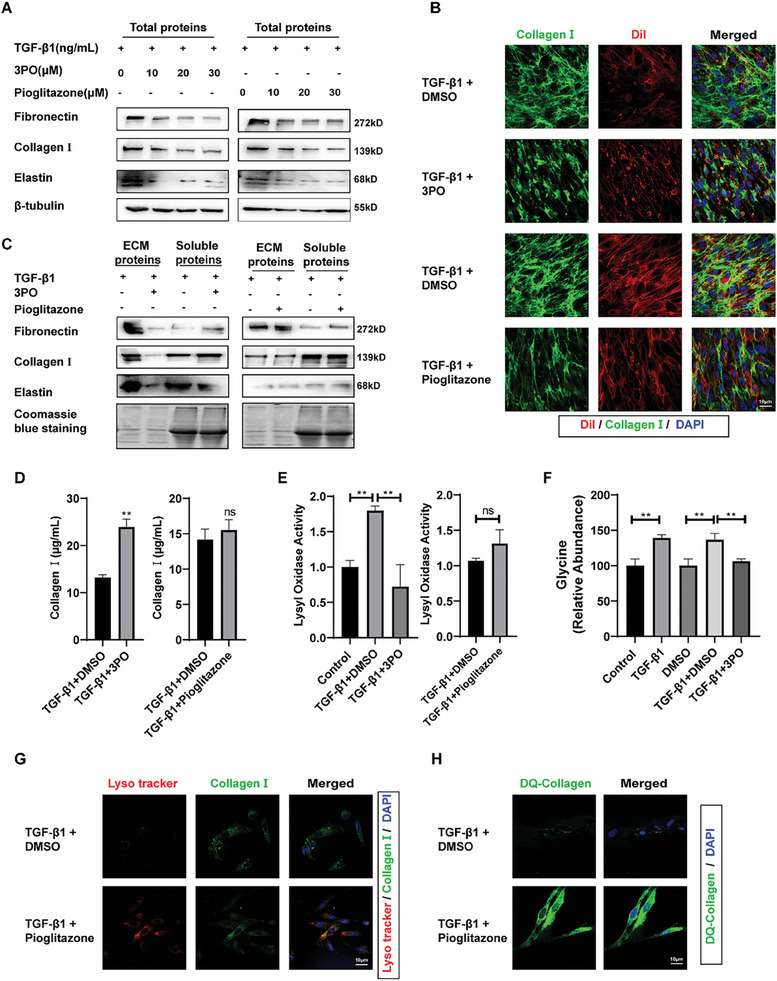
Inhibiting glycolysis or enhancing FAO affects ECM deposition in activated fibroblasts. A) Western blot analysis for total protein levels of core components of ECM in MRC‐5 cells co‐treated with TGF‐β1 and 3PO or pioglitazone. The results of the experiment were repeated at least 3 times. B) Representative image showing collagen I (green) in MRC‐5 cells. Cell membranes were stained with DiI (red) and nuclei were stained with DAPI (blue). C) Western blot analysis for the levels of ECM molecules in extracellular and soluble proteins. Each experiment was performed in triplicate to ensure reproducibility of results. D) The content of soluble collagen in cell culture media detected by Sircol soluble collagen assay kit. All data were expressed as the means ± SD of at least 3 independent experiments, ^*^ *p* < 0.05 and ^**^ *p* < 0.01. E) Lysyl oxidase activity in MRC‐5 cells co‐treated with TGF‐β1 and 3PO or pioglitazone. All data were expressed as the means ± SD of at least 3 independent experiments, ^*^ *p* < 0.05 and ^**^ *p* < 0.01. F) The effects of the TGF‐β1 and 3PO on relative abundance of glycine in MRC‐5 cells. All data were expressed as the means ± SD of at least 3 independent experiments, ^*^ *p* < 0.05 and ^**^ *p* < 0.01. G) Colocalization of lysosome and collagen I in MRC‐5 cells. MRC‐5 cells were subjected to immunofluorescence analysis with lyso tracker (red) and anti‐ collagen I (green). Nuclei are stained with DAPI (blue). H) Representative images of DQ‐collagen internalized by MRC‐5 cells. Nuclei are stained with DAPI (blue).

To investigate the cellular metabolic change in fibroblasts, we modified the metabolic conditions of MRC‐5 cells by inhibiting glycolysis and FAO, respectively. Inhibition of glycolysis with 3‐(3‐pyridinyl)‐1‐(4‐pyridinyl)‐2‐propen‐1‐one (3PO) or enhancement of FAO with pioglitazone both resulted in reduced expression of ECM‐related proteins in TGF‐β1‐treated fibroblasts (**Figure** **3**A; Figure S3A, Supporting Information). Decreased expression of collagen I in activated fibroblasts was consistently shown through immunofluorescence staining after administering 3PO and pioglitazone (Figure S3B, Supporting Information). Notably, we observed that the inhibition of glycolysis by using 3PO resulted in the reduction of extracellular haphazard collagen I, while intracellular fluorescence signals maintained their strength. Conversely, enhancement of FAO by using pioglitazone resulted in extracellular fluorescence signals remaining of high intensity but the intracellular signals for collagen decreased relatively. To enhance the visual representation of this phenomenon, we utilized DiI fluorescent dye to label the cell membrane, allowing us to distinguish between collagen inside and outside the cell. Consistent with previous findings, inhibiting glycolysis and increasing FAO resulted in distinct alterations in collagen within and outside the cell (Figure 3B, Supporting Information). These findings suggest that these 2 metabolic interventions may influence collagen deposition through different mechanisms.

Next, we investigated how modified metabolic conditions impact ECM deposition through various facets of ECM synthesis, modification, and degradation in activated fibroblasts. Inhibition of glycolysis was followed by a decrease in insoluble ECM, whereas enhancement of FAO did not change significantly, as shown by western blot and immunofluorescence results (Figure 3C; Figure S3C, Supporting Information). Meanwhile, the Sircol assay demonstrated an increase in soluble collagen in the culture medium after 3PO treatment, whereas pioglitazone treatment had no significant effect (Figure 3D). The cross‐linking of structural components of the ECM, in particular fibrillar collagens and elastin, is strongly associated with the progression of fibrosis and its resistance to reversal. Members of the Lysyl oxidase (LOX) family are copper‐dependent enzymes located extracellularly, fulfilling a crucial role in cross‐linking ECM, thus leading to less vulnerable ECM degradation. Thus, we investigated whether variations in collagen solubility resulted from changes in ECM cross‐linking modifications. Reducing glycolysis inhibited the elevation of LOX family enzyme activity induced by TGF‐β1, while increasing FAO did not show a significant effect (Figure 3E). In addition, hindering glycolysis significantly lowered the quantity of glycine, a key component in the production of collagen (Figure 3F).

Collagen degradation is regulated not only by the extracellular degradation pathway but also by the intracellular pathway through internalization and subsequent lysosomal degradation. To further assess the intracellular pathway of collagen degradation after metabolic intervention, we utilized intracellular lysosome fluorescence labelling to determine the degree of intensity of intracellular degradation. The results indicate that enhancing FAO increases lysosomal signal intensity and co‐localization with collagen (Figure S3D,G, Supporting Information). The introduction of exogenous DQ‐collagen provides additional evidence that pioglitazone treatment augments the uptake of collagen and subsequent degradation pathways within fibroblasts (Figure 3H). Our findings collectively indicate that the promotion of FAO and the suppression of glycolysis effectively impede the deposition of ECM. Nevertheless, it is noteworthy that the mechanisms underlying these inhibitory processes differ, leading to varying effects.

### 2.4 Interventional and Therapeutic Effects of Inhibiting Glycolysis and Enhancing FAO In Vivo

**Figure 4:**
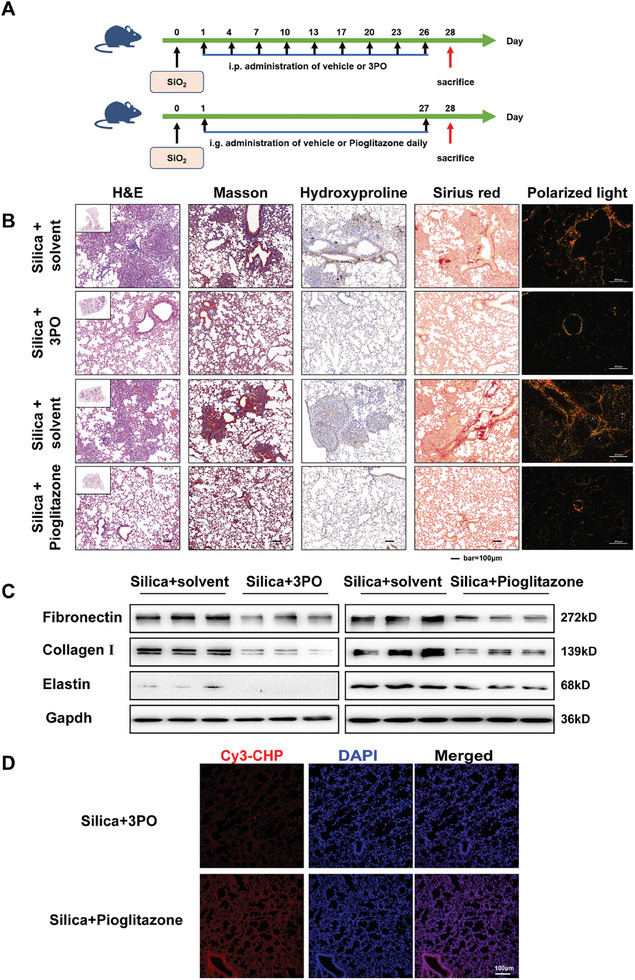
Inhibiting glycolysis or enhancing FAO impedes the progression of fibrosis in vivo. A) Schematic outline of the 3PO or pioglitazone intervention model in mice with silica‐induced lung fibrosis. B) Histological analysis was conducted on mouse lung tissue using H&E staining (first image from left), Masson staining (second image from left), Hydroxyproline immunohistochemical staining (third image from left), Sirius red staining (second image from right), and polarized light imaging (first image from right); scale bar = 100 µm. C) The protein levels of fibronectin, collagen I, and elastin in each group were examined by the western blot. The results of the experiment were repeated at least 3 times. D) Representative fluorescence micrographs of fibrotic lung areas were stained with fluorescently labelled collagen hybridisation peptide (Cy3‐CHP). Nuclei are stained with DAPI (blue). Selected micrographs are representative of images collected from each group.

Based on the above, interventions that target fibroblast metabolic perturbations may reduce ECM deposition and attenuate pulmonary fibrosis. Further, to understand whether metabolic regulator can improve fibrotic remodeling and is effective in attenuating lung fibrosis, our group developed intervention models for mice with silica dust‐induced pulmonary fibrosis using the glycolysis inhibitor 3PO and the FAO activator pioglitazone, respectively (**Figure** **4**A). Staining of pathological sections revealed that intervention with 3PO or pioglitazone immediately after silica dust exposure significantly reduced alveolar structural disruption and pulmonary fibrosis (Figure 4B). Collagen deposition was significantly reduced after 28 days of silica dust treatment, as demonstrated by Masson and Sirius red staining. However, there was still a notable presence of hydroxyproline staining following 3PO intervention, as shown by immunohistochemical staining with a specific antibody for hydroxyproline (Figure 4B). Moreover, the expression of fibronectin, collagen I and elastin were downregulated in the lung tissues of silicosis mice following interventions with 3PO and pioglitazone (Figure 4C), suggesting that the enhancement of FAO and inhibition of glycolysis can mitigate silica dust‐induced pulmonary fibrosis to differing extents. The synthetic peptide known as Collagen heterotrimeric peptide (CHP) exhibits specific binding affinity to denatured collagen molecules. In mouse lung tissue subjected to intervention with 3PO, the fluorescence intensity of CHP was observed to be low, whereas in the pioglitazone groups, the fluorescence intensity of CHP was notably high and localized around the nucleus (Figure 4D). In contrast to hydroxyproline immunohistochemical staining, the collagen stained by CHP did not appear to be fully degraded.

**Figure 5:**
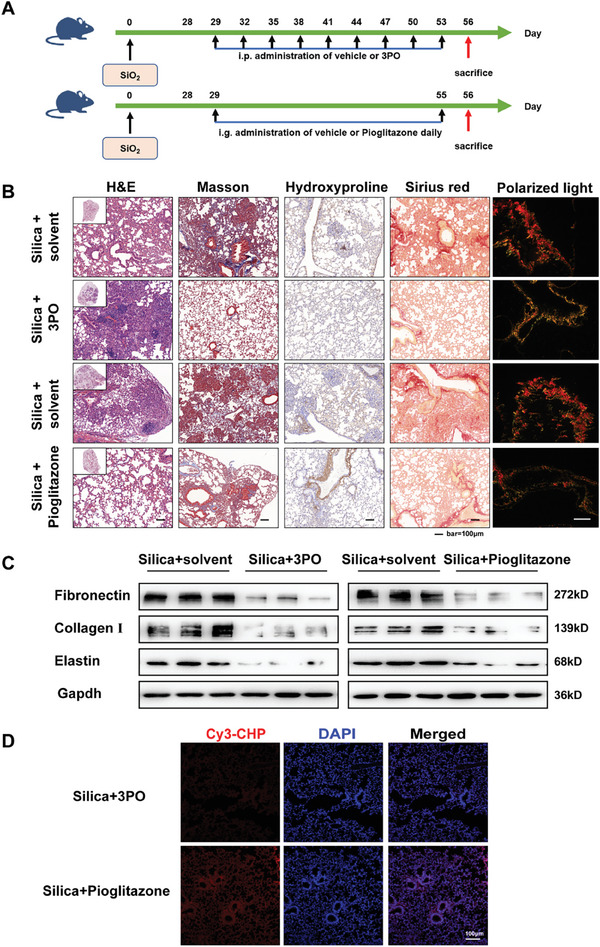
The administration of 3PO or pioglitazone has been demonstrated to provide therapeutic relief for established pulmonary fibrosis in vivo. A) Schematic outline of the 3PO or pioglitazone treatment model in mice with silica‐induced lung fibrosis. B) Histological analysis was conducted on mouse lung tissue using H&E staining (first image from left), Masson staining (second image from left), Hydroxyproline immunohistochemical staining (third image from left), Sirius red staining (second image from right), and polarized light imaging (first image from right), scale bar = 100 µm. C) The protein levels of fibronectin, collagen I, and elastin in each group were examined by the western blot. The results of the experiment were repeated at least 3 times. D) Representative fluorescence micrographs of fibrotic lung areas were stained with fluorescently labelled collagen hybridisation peptide (Cy3‐CHP). Nuclei are stained with DAPI (blue). Selected micrographs are representative of images collected from each group.

Then, we assessed the therapeutic effect of 2 metabolic modulators on established pulmonary fibrosis (**Figure** **5**A). Histopathological analysis of mouse lung tissue revealed that treatment with pioglitazone partially mitigated lung fibrosis, although collagen deposits persisted. In contrast, administration of 3PO significantly attenuated lung damage, yet hydroxyproline‐rich collagen fragments remained present in the tissue (Figure 5B). The results of western blot indicate that metabolic modulator treatments led to a reduction in the expression of fibronectin, collagen I, and elastin in lung tissue (Figure 5C). The results of CHP staining paralleled those observed in the intervention group, indicating that, in the context of pulmonary fibrosis, the enhancement of FAO did not facilitate the complete degradation of fibrotic ECM (Figure 5D). These findings indicate that the inhibition of glycolysis and enhancement of FAO have varying effects on the progression and establishment of silica dust‐induced pulmonary fibrosis in mice. Nevertheless, the mechanisms underlying these improvements seem to be incongruent.

### 2.5 HIF‐1α Regulated the Transformation of FAO‐Glycolysis in Fibroblast

**Figure 6:**
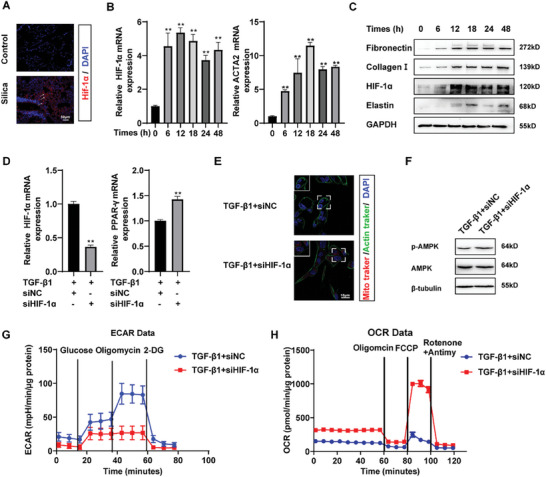
HIF‐1α regulated the transformation of FAO‐glycolysis in fibroblast. A) Representative image showing HIF‐1α (red) stained with anti‐HIF‐1α antibody in mouse lung tissue. Nuclei were stained with DAPI (blue). B) Relative expression of HIF‐1α and α‐SMA mRNA in MRC‐5 cells at different time‐points after 5ng/mL TGF‐β1 treatment by qRT‐PCR. All data were expressed as the means ± SD of at least 3 independent experiments, ^*^ *p* < 0.05 and ^**^ *p* < 0.01. C) The protein levels of fibronectin, collagen I, HIF‐1α, and elastin in MRC‐5 cells at different time‐points after 5ng/mL TGF‐β1 treatment were examined by the western blot. The results of the experiment were repeated at least 3 times. D) Relative expression of HIF‐1α and PPAR‐γ mRNA in MRC‐5 cells transfected with siHIF‐1α by qRT‐PCR. All data were expressed as the means ± SD of at least 3 independent experiments, ^*^ *p* < 0.05 and ^**^ *p* < 0.01. E) Mitochondrial content was measured by Mito tracker red after transfected with siHIF‐1α, Actin tracker (green) labelling of cellular microfilaments. Nuclei were stained with DAPI (blue). F) Western blot analysis for protein levels of phosphorylated AMPK and AMPK in MRC‐5 cells. The results of the experiment were repeated at least 3 times. G) and H) Real‐time measurements to determine the effect of knockdown HIF‐1α on ECAR and OCR in MRC‐5 cells.

In this study, we discovered that the inhibition of glycolysis or the enhancement of FAO can mitigate fibrotic ECM deposition via distinct pathways. Cell metabolism experiments revealed that the inhibition of glycolysis using 3PO led to an increase in the OCR (Figure S4A, Supporting Information). Conversely, enhancing FAO with pioglitazone resulted in only a minimal reduction in the ECAR in cells (Figure S4B, Supporting Information). This suggests that the metabolic alterations occurring when glycolysis or fatty acid oxidation is inhibited in isolation are complex and unpredictable. Therefore, it is imperative to identify a key regulatory factor that modulates both metabolic pathways. In our transcriptomic data, we observed an increase in HIF‐1α, a protein closely associated with metabolic regulation and which is implicated in several diseases.^[^ ^16^ ^]^ Immunohistochemical staining of lung tissue from patients with IPF and silicosis revealed an upregulation of HIF‐1α in fibrotic lung tissue and a downregulation of (peroxisome proliferator‐activated receptor‐gamma) (PPAR‐γ) (Figure S5A, Supporting Information). Further testing in mice with silica dust‐induced lung fibrosis confirmed the previous findings, as there was a significant increase in HIF‐1α expression observed in silicosis nodules (**Figure** **6**A). Based on the previously constructed model of TGF‐β1‐induced fibroblast activation, we examined the mRNA and protein levels of HIF‐1α to determine whether it functions in fibroblasts. Our results indicate that both mRNA and protein levels were elevated (Figure 6B,C; Figure S5B, Supporting Information). Treatment with TGF‐β1 delayed the degradation of HIF‐1α protein after inhibiting protein synthesis using cycloheximide (CHX) (Figure S5D, Supporting Information). Furthermore, the results of IP experiments indicate that the ubiquitination level of HIF‐1α decreased following TGF‐β1 treatment (Figure S5E, Supporting Information). These findings suggest that TGF‐β1 enhances the expression and stabilization of HIF‐1α in fibroblasts under normoxic conditions.

Further assays were performed to determine whether HIF‐1α regulates the metabolic transition from glycolysis to fatty acid oxidation in TGF‐β1‐induced activated fibroblasts. The results of using specific siRNA to knock down HIF‐1α in MRC‐5 cells (Figure 6D) and detecting mitochondrial alterations showed that the reduction of HIF‐1α reversed the mitochondrial number (Figure 6E). Furthermore, the knockdown of HIF‐1α restored AMPK activity (Figure 6F; Figure S5C, Supporting Information) and increased the expression of PPAR‐γ (Figure 6D). The cellular metabolism assay demonstrated that the knockdown of HIF‐1α inhibited the TGF‐β1‐induced elevation of glycolysis while restoring the fatty acid oxidation level (Figure 6G–H). The results indicate that HIF‐1α mediates the fatty acid oxidation‐glycolysis metabolic shift in TGF‐β1‐induced activated fibroblasts.

### 2.6 HIF‐1α Inhibition Reduced ECM Deposition by Regulating Glycolytic‐FAO Metabolic Disturbance

**Figure 7:**
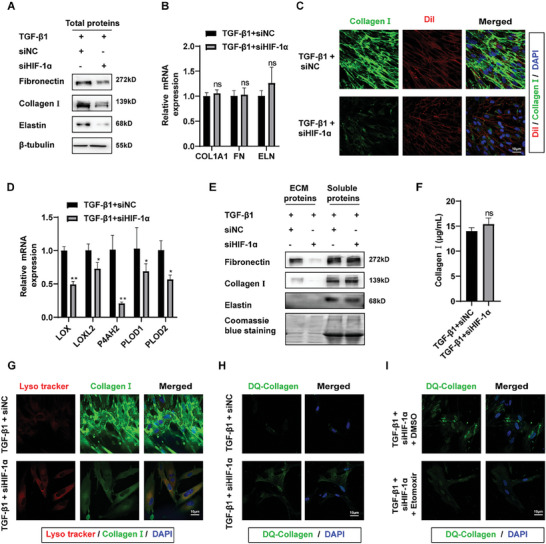
HIF‐1α inhibition reduced ECM deposition by regulating glycolytic‐FAO metabolic disturbance. A) Western blot analysis for total protein levels of core components of ECM in MRC‐5 cells co‐treated with HIF‐1α siRNA and TGF‐β1. The results of the experiment were repeated at least 3 times. B) Relative expression of core components of ECM in MRC‐5 cells co‐treated with HIF‐1α siRNA and TGF‐β1 by qRT‐PCR. All data were expressed as the means ± SD of at least 3 independent experiments, ^*^ *p* < 0.05 and ^**^ *p* < 0.01. C) Representative image showing collagen I (green) in MRC‐5 cells. Cell membranes were stained with DiI (red) and nuclei were stained with DAPI (blue). D) qRT‐PCR analysis for the mRNA expression of some ECM‐modifying enzymes (LOX, LOXL2, P4AH2, PLOD1, PLOD2) in MRC‐5 cells co‐treated with HIF‐1α siRNA and TGF‐β1. All data were expressed as the means ± SD of at least 3 independent experiments, ^*^ *p* < 0.05 and ^**^ *p* < 0.01. E) Western blot analysis for the levels of ECM molecules in extracellular and soluble proteins. Each experiment was performed in triplicate to ensure reproducibility of results. F) The content of soluble collagen in cell culture media detected by Sircol soluble collagen assay kit. All data were expressed as the means ± SD of at least 3 independent experiments, ^*^ *p* < 0.05 and ^**^ *p* < 0.01. G) Colocalization of lysosome and collagen I in MRC‐5 cells. MRC‐5 cells were subjected to immunofluorescence analysis with lyso tracker (red) and anti‐ collagen I (green). Nuclei are stained with DAPI (blue). H) and I) Representative images of DQ‐collagen internalized by MRC‐5 cells. Nuclei are stained with DAPI (blue). LOXL2: lysyl oxidase like 2; P4AH2: prolyl 4‐hydroxylase 2; PLOD1: procollagen‐lysine,2‐oxoglutarate 5‐dioxygenase 1; PLOD2: procollagen‐lysine,2‐oxoglutarate 5‐dioxygenase 2.

Is it feasible to achieve more effective removal of fibrotic ECM by concurrently modulating metabolic flow, considering that inhibition of glycolysis and enhancement of FAO operate through distinct pathways and possess individual merits and drawbacks? To investigate whether inhibiting HIF‐1α expression can more effectively scavenge excess ECM by modulating glycolysis and fatty acid oxidation metabolic perturbations, we downregulated its expression in MRC‐5 cells and analyzed ECM molecular levels. Knockdown of HIF‐1α in MRC‐5 cells led to a decrease in the expression of ECM‐related molecules (**Figure** **7**A; Figure S6A, Supporting Information). Additionally, immunofluorescence results indicated the fluorescence intensity of both intracellular and extracellular collagen I was significantly reduced after HIF‐1α knockdown (Figure 7C; Figure S6B, Supporting Information). Fluorescence staining of the ECM after decellularization also revealed a decrease in collagen content (Figure S6C, Supporting Information). These results demonstrate that inhibiting HIF‐1α could effectively reduce TGF‐β1‐induced ECM deposition in vitro.

Based on these, we further investigated whether inhibiting HIF‐1α resulted in a reduction of ECM through the inhibition of glycolysis and fatty acid oxidation metabolic perturbations. As HIF‐1α is a transcription factor with broad effects, we analyzed the transcriptional impact on ECM molecules following HIF‐1α knockdown. The study found that the transcriptional levels of ECM molecules such as collagen remained unchanged upon knockdown of HIF‐1α (Figure 7B). However, the transcriptional levels of ECM‐modifying enzymes (LOX, LOXL2, P4AH1, PLOD1, and PLOD2) were reduced (Figure 7D). The experimental results showed that inhibition of HIF‐1α, similar to inhibition of glycolysis and activation of fatty acid oxidation, led to changes in ECM solubility and fibroblast internalization (Figure 7E–I). HIF‐1α can affect ECM modification through its own transcriptional level and ECM deposition by regulating the metabolic shift from fatty acid oxidation to glycolysis.

### 2.7 Inhibition of HIF‐1α‐Regulated FAO‐Glycolysis Metabolic Perturbation Attenuates Pulmonary Fibrosis In Vivo

**Figure 8:**
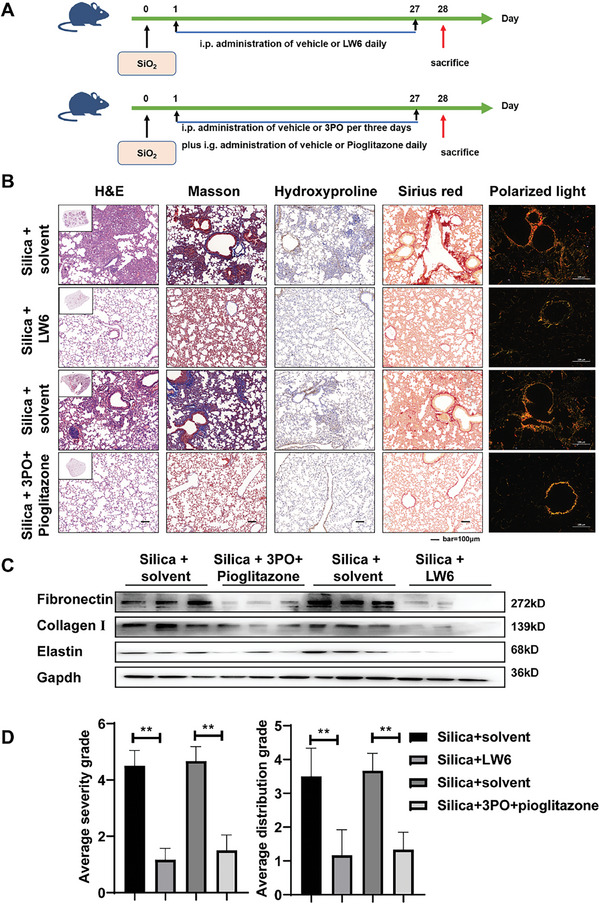
Inhibition of HIF‐1α or FAO‐glycolysis metabolic perturbation impedes the progression of fibrosis in vivo. A) Schematic outline of the intervention model in mice with silica‐induced lung fibrosis. B) Histological analysis was conducted on mouse lung tissue using H&E staining (first image from left), Masson staining (second image from left), Hydroxyproline immunohistochemical staining (third image from left), Sirius red staining (second image from right), and polarized light imaging (first image from right); scale bar = 100 µm. C) The protein levels of fibronectin, collagen I, and elastin in each group were examined by the western blot. D) Severity of fibrosis score and distributions of fibrosis grade in mice (*n* = 6), ^**^ *p* < 0.01.

**Figure 9:**
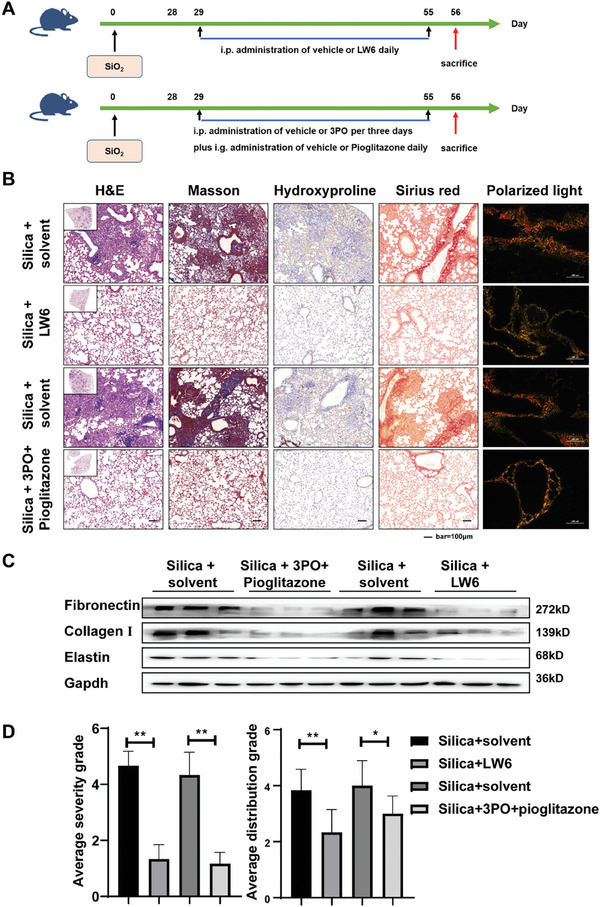
Inhibition of HIF‐1α or improvement of FAO‐glycolysis metabolic perturbation promotes regression of established fibrosis in vivo. A) Schematic outline of the treatment model in mice with silica‐induced lung fibrosis. B) Histological analysis was conducted on mouse lung tissue using H&E staining (first image from left), Masson staining (second image from left), Hydroxyproline immunohistochemical staining (third image from left), Sirius red staining (second image from right), and polarized light imaging (first image from right); scale bar = 100 µm. C) The protein levels of fibronectin, collagen I, and elastin in each group were examined by the western blot. D) Severity of fibrosis score and distributions of fibrosis grade in mice (*n* = 6), ^*^ *p* < 0.05 and ^**^ *p* < 0.01.

Finally, we investigated whether targeting HIF‐1α and its associated metabolic changes could be a potential treatment for pulmonary fibrosis. On the second day after silica dust titration or on day 29, mice were treated with a combination of 2 metabolic modulators (3PO plus pioglitazone) or a HIF‐1α inhibitor (LW6), respectively, to assess intervention or treatment effects (**Figures** **8**A and **9**A). We performed a drug safety evaluation on mice designated as the intervention group. The findings indicated that there was no significant reduction in body weight (Figure S7A, Supporting Information), no notable elevation in serum alanine aminotransferase (ALT) and aspartate aminotransferase (AST) levels (Figure S7B, Supporting Information), and no significant pathological alterations in hepatic and renal tissues (Figure S7C, Supporting Information). These results suggest that the combination of LW6 and 3po with pioglitazone does not produce significant adverse effects in mice. The H&E staining of mouse lung tissues revealed a reduction in fibrosis in the lungs of mice in the LW6 group and the 3PO combined with pioglitazone group. Additionally, Masson staining and hydroxyproline immunohistochemistry demonstrated a relative decrease in collagen deposition in the lung tissues of mice. Meanwhile, the staining of Sirius scarlet showed a decrease in collagen deposition, and polarised light microscopy revealed a reduction in the complex and tightly packed collagen in the LW6 group and the 3PO combined with pioglitazone group. (Figures 8B and 9B). Western blot results showed the expression of fibronectin, collagen I and elastin were downregulated in the LW6 group and the 3PO combined with pioglitazone group (Figures 8C and 9C). Consistent with this, we found evident improvement in the severity and location of fibrotic lesions (Figures 8D and 9D). These results suggest that inhibiting HIF‐1α‐regulated metabolic shifts in vivo can effectively reduce the excessive deposition of ECM in the lungs of mice exposed to silica dust.

## 3 Discussion

The prolonged exposure to silica‐rich dust during occupational duties can result in the onset of silicosis, characterized by persistent lung inflammation, alveolar structure deterioration, silica nodule formation, and, in severe instances, extensive fibrosis of pulmonary tissue. The development of innovative work environments introduces the emergence of new scenarios for exposure to silica dust.^[^ ^25^ ^]^ The pathogenesis of silicosis is intricate and remains incompletely understood, posing a challenge for therapeutic drug development. A comprehensive investigation of the underlying mechanisms of silicosis is essential for the creation of effective therapeutic strategies.

Fibrosis is distinguished by the excessive accumulation of ECM, causing disruption to tissue organization and functionality. In a healthy pulmonary system, the ECM not only governs tissue organization but also offers mechanical support and elastic recoil, crucial for proper lung function. Conversely, in fibrotic lung tissue, alterations in the composition and characteristics of the ECM contribute to abnormal lung function.^[^ ^4^ ^]^ Myofibroblasts, as the primary effector cells responsible for ECM production, are derived from various precursor cells activated by TGF‐β1. Numerous studies have demonstrated the efficacy of inhibiting or reversing fibroblast activation in reducing the accumulation of fibrotic ECM.^[^ ^17^ ^]^ Nevertheless, their lack of specificity in targeting myofibroblasts may result in various complications. Additionally, TGF‐β1, a key mediator of fibrosis, plays a role in suppressing inflammation and excessive epithelial proliferation during tissue repair to maintain tissue homeostasis.^[^ ^18^ ^]^ Regrettably, suppression of TGF‐β1 can lead to negative consequences. While improving effectiveness and reducing adverse reactions, the presence of various surface proteins is not limited to myofibroblasts,^[^ ^19^ ^]^ such as cadherin‐11^[^ ^20^ ^]^ and platelet‐derived growth factor (PDGF) receptors α.^[^ ^21^ ^]^ Consequently, directly targeting fibrotic ECM presents a more appealing potential strategy. The excessive deposition of ECM is not solely a result of fibrosis but also a pivotal factor in the progression of disease.^[^ ^3^, ^22^ ^]^ In fibrotic diseases, dysregulated ECM turnover and overproduction contribute to sustained aberrant signaling. Studies have demonstrated that decellularized lung ECM derived from individuals with IPF can prompt the differentiation of lung fibroblasts into myofibroblasts without exogenous cytokines.^[^ ^23^ ^]^ Furthermore, the elevated stiffness of lung tissue and ECM in pulmonary fibrosis led to the e development of a pro‐fibrotic feedback loop.^[^ ^24^ ^]^ The progression of renal fibrosis is exacerbated by the mechanosensitive non‐selective cation channel Piezo1.^[^ ^25^ ^]^ To address pulmonary fibrosis and improve lung function, it is crucial to facilitate the degradation of fibrotic ECM and encourage the regeneration of physiological ECM. However, this field of investigation presents significant challenges. Our study delved into the molecular pathways implicated in ECM restructuring in pulmonary fibrosis induced by silica dust. Concretely, we investigated the disruption of glycolysis‐FAO metabolism to modulate ECM remodeling and identified potential metabolic targets for the elimination of fibrotic ECM through synthesis, modification, and degradation pathways.

Our findings demonstrated that fibrotic lung tissues induced by silica exposure showed increased expression of genes involved in the regulation of glycolysis, as well as elevated levels of metabolites associated with this metabolic pathway. Conversely, there was a reduction in the expression of genes related to the regulation of FAO and a corresponding decrease in associated metabolites. These alterations in cellular metabolism, particularly in glycolysis and FAO, have been identified as significant mechanisms contributing to various pathological processes. Metabolomics analysis has further demonstrated the metabolic alterations in various respiratory conditions such as cystic fibrosis, asthma, and chronic obstructive pulmonary disease (COPD).^[^ ^6^, ^26^ ^]^ Numerous investigations have confirmed the intimate relationship between aerobic glycolysis and pulmonary fibrosis,^[^ ^26c^ ^]^ as well as the identification of certain lipid molecules in plasma as potential biomarkers for IPF.^[^ ^27^ ^]^ These findings support the conclusions of the present study, suggesting that targeting metabolic dysregulation is imperative for the management of pulmonary fibrosis and may offer a viable strategy for improving the elimination of fibrotic ECM.

In our investigation, we have demonstrated that the stimulation of lung fibroblasts is linked to heightened glycolytic activity to fulfill their requirements for growth and secretion. The intermediary metabolites generated during glycolysis serve as crucial biosynthetic precursors that facilitate support processes such as protein synthesis and cellular proliferation. Despite producing lower ATP than OXPHOS, glycolysis is characterized by faster reaction rates and the production of numerous biosynthetic intermediates. The increased requirement for protein synthesis and biosynthetic intermediates is a prominent feature of fibrosis. In vitro studies have shown that inhibiting glycolysis, either by inhibiting glucose transport proteins or key enzymes in the glycolytic pathway, can hinder fibroblast differentiation and ECM production.^[^ ^9^, ^28^ ^]^ Previous studies have demonstrated the potential of glycolysis to reverse fibrosis in vivo, specifically in cases such as bleomycin‐induced pulmonary fibrosis.^[^ ^29^ ^]^ Additionally, glycolysis plays a role in providing amino acids for collagen synthesis, with 3‐phosphoglycerate serving as a precursor for serine, which is subsequently converted to glycine.^[^ ^30^ ^]^ This amino acid is crucial for the stabilization of the collagen helix. Our research findings align with the notion that inhibiting glycolysis using 3PO in case of silicosis leads to a reduction in glycine levels in fibroblasts, ultimately resulting in decreased collagen synthesis.

The final products of glycolysis may contribute to the stability and hydroxylation of collagen.^[^ ^11^ ^]^ In this study, we assessed the effects of glycolysis inhibition on ECM modification and revealed that inhibiting glycolysis led to a reduction in the enzymatic activity of the LOX family, a crucial enzyme responsible for cross‐linking collagen and elastin. Consequently, tissue stiffness increased in fibrotic conditions, potentially hindering collagen degradation and promoting the accumulation of extracellular collagen. Subsequent study has demonstrated that downregulation of HIF‐1α does not affect the transcription levels of ECM molecules, specifically collagen. However, it does lead to decreased mRNA levels of ECM modification‐related molecules, including LOX and PLOD family members. Furthermore, HIF‐1α knockdown has been shown to impede the activity of LOX family enzymes. These results indicate that suppression of glycolysis and targeting HIF‐1α could effectively diminish both ECM synthesis and modification, offering a promising strategy for mitigating fibrotic ECM deposition. Xie et al discovered that the glycolysis inhibitor 3PO effectively alleviated bleomycin‐induced pulmonary fibrosis and decreased the levels of collagen, fibronectin, and α‐SMA.^[^ ^31^ ^]^ However, results from hydroxyproline immunohistochemical staining experiments conducted on mouse lung tissue sections indicated that hydroxyproline deposition persisted in the lungs of mice in the 3PO‐treated group. Despite the distinct characteristics of bleomycin‐ and silica dust‐induced pulmonary fibrosis, it is hypothesized that the persistence of hydroxyproline after 3PO intervention may be due to incomplete clearance of the compound from tissues following collagen degradation. The biological activity of extracellular matrix degradation products is commonly acknowledged. Hence, current interventions and treatment strategies utilizing 3PO to enhance ECM degradation for fibrosis mitigation are not yet fully developed. This highlights the importance of simultaneously addressing the promotion of fibrotic ECM degradation and the elimination of degradation byproducts when formulating strategies to reverse fibrosis.

Our research investigated the correlation between FAO and fibrosis, aligning with previous studies indicating compromised FAO in diverse fibrotic conditions. This implies a connection between fibrosis and increased fatty acid synthesis coupled with decreased FAO. Alterations of the levels of saturated and unsaturated fatty acids have been observed in fibrosis patients and animal fibrosis models. Patients with IPF exhibited a 63% increase in serum total fatty acid concentrations.^[^ ^32^ ^]^ In pulmonary fibrosis, a decrease in the activity of AMPK, a crucial regulator of cellular metabolism, has been observed. AMPK is involved in promoting fatty acid oxidation and inhibiting fatty acid synthesis by phosphorylating acetyl‐CoA carboxylase 1 (ACC1) and sterol regulatory element‐binding protein 1c (SREBP1c).^[^ ^33^ ^]^ Our previous study has demonstrated that metformin activates AMPK in fibroblasts, thereby attenuating the progression of silicosis.^[^ ^34^ ^]^ The PPAR signaling pathway serves as a significant regulator of FAO, with peroxisome proliferator‐activated receptors (PPARs) comprising a family of transcription factors consisting of 3 major isoforms (α, δ, and γ) that exhibit varying tissue expression patterns and distinct functions in lipid metabolism. Numerous studies have shown that fibrotic tissues commonly display diminished PPAR signaling in response to TGF‐β1, leading to a decline in FAO.^[^ ^35^ ^]^ Analysis of the transcriptome in mice with silica dust‐induced lung fibrosis revealed the downregulation of PPAR‐α and PPAR‐γ. Subsequent validation experiments confirmed a relatively higher abundance of PPAR‐γ in lung tissue, suggesting a potential role for PPAR‐γ as a key regulator of FAO in lung fibroblasts. A study revealed a decrease in cluster of differentiation 36 (CD36), a protein involved in fatty acid transport and PPAR signaling, in both human and murine dermal fibrosis, further supporting the association between FAO and extracellular matrix regulation.^[^ ^13^ ^]^ Consistent with this research, our study demonstrated that pioglitazone, an activator of FAO, promoted the resolution of silica‐induced lung fibrosis by facilitating ECM internalization by myofibroblasts. These findings advance the current knowledge of fatty acid oxidation in the pathogenesis of pulmonary fibrosis.

The collagen molecule is distinguished by its triple helix structure, resulting from the arrangement of 3 linear protein chains into a trimeric conformation. Upon cleavage by proteases, such as matrix metalloproteinases, the collagen molecule undergoes denaturation and unfolding. CHP is a synthetic peptide that exhibits selective binding to the degraded and denatured collagen molecule, while showing no affinity for intact collagen.^[^ ^36^ ^]^ Our findings suggest that the fluorescent signal of CHP was notably reduced in lung tissues treated with 3PO as compared to those treated with pioglitazone in both the intervention and treatment models. This contrasts with hydroxyproline staining, which detects fully hydrolyzed collagen products, while CHP specifically binds to denatured collagen strands by re‐forming a triple‐helical structure. Collagen degradation is mediated via both extracellular and intracellular pathways. The intracellular pathway entails the binding and uptake of collagen fragments by fibroblasts or macrophages, followed by their degradation within lysosomes. In bleomycin‐induced mouse lung fibrosis, mice lacking the receptor responsible for collagen ingestion showed an increase in total lung hydroxyproline content without changes in inflammation or collagen synthesis rates.^[^ ^37^ ^]^ Consequently, in alignment with our cellular experiments, the augmentation of FAO appears to facilitate the turnover of collagen degradation products in the context of collagen breakdown. This effect may be partially attributed to the enhancement of intracellular degradation pathways by increased FAO.

In vivo, it was observed that the metabolic modulators, 3PO and pioglitazone, yielded divergent results. The pioglitazone intervention, which increases FAO, not only exhibited beneficial intervention effect but also significantly reduced hydroxyproline levels in lung tissue. This implies that hindering FAO may impede fibrosis progression while aiding in the removal of collagen degradation products. However, the results of the pathological section staining indicate that the effectiveness of the pioglitazone treatment model was comparatively limited. This indicates that simply increasing FAO may not be sufficient in completely eradicating the fibrotic ECM. A study conducted in 2009 demonstrated that pioglitazone was able to prevent fibrotic alterations and the increase in hydroxyproline levels in the lungs following BLM titration.^[^ ^38^ ^]^ Our study contributes to the existing body of evidence that suggests pioglitazone has the potential to attenuate pulmonary fibrosis by enhancing FAO in the initial phases of injury‐induced fibrosis, particularly in cases of early exposure to silica dust. It is crucial to acknowledge that solely augmenting FAO may not be sufficient for managing established pulmonary fibrosis. Cellular metabolism is a complex network that adapts to local substrate availability to fulfil cellular requirements for energy, redox balance and biomass generation. The metabolic wiring of cells is not only determined by energy requirements, but also by biosynthetic needs. Emerging studies have found that the metabolic shift between glycolysis and FAO regulates cell differentiation, proliferation, migration and other activities.^[^ ^39^ ^]^ The previous study demonstrated that the perturbation of glycolysis and FAO affects the fibroblast‐to‐myofibroblast transition, and metabolic dysregulation is an exciting new therapeutic target to treat pulmonary fibrosis.^[^ ^7b^ ^]^ These studies indicate that regulating the metabolic flux between FAO and glycolysis could potentially be a target for disease treatment.

HIF‐1α typically responds to hypoxic conditions and modulates cellular energy metabolism. However, in normoxic conditions, proline residues located in the oxygen‐dependent degradation domain (ODDD) of HIF‐1α undergo hydroxylation by prolyl hydroxylase (PHD), leading to the rapid degradation of hydroxylated HIF‐1α by the ubiquitin‐proteasome system.^[^ ^40^ ^]^ Recent studies have revealed that non‐hypoxic stimuli, including lipopolysaccharide (LPS), thrombin, and Ang II, can elevate and stabilize HIF‐1α protein levels.^[^ ^41^ ^]^ This study demonstrated that TGF‐β1 treatment of fibroblasts resulted in a notable increase in HIF‐1α mRNA levels and a reduction in protein ubiquitination, suggesting that TGF‐β1 induces an increase in the level of HIF‐1α and stabilizes its protein level under normoxic conditions.

Our study showed that HIF‐1α plays a crucial role in modulating metabolic alterations in silica dust‐induced pulmonary fibrosis. By regulating the shift of FAO and glycolysis, HIF‐1α facilitates the degradation of fibrotic ECM and mitigates fibrosis progression. In vitro experiments further substantiate these findings, revealing that suppression of HIF‐1α impedes the reduction of fibroblast glycolysis levels induced by TGF‐β1, while restoring FAO levels. Moreover, HIF‐1α knockdown decrease ECM synthesis and modification, as well as enhances the internalization of ECM by fibroblasts. These results diverge from prior research that identified a function for HIF‐1α in the regulation of fibroblast activation.^[^ ^7b^ ^]^ Lung fibrosis, particularly silicosis, is commonly diagnosed in the later stages of fibrosis development. Merely inhibiting or reversing fibroblast activation at this point does not comprehensively address the fibrosis. Removal of the fibrotic ECM is essential for the restoration of lung function. Our findings support the significance of HIF‐1α as a molecular target for the reversal of pulmonary fibrosis and provide valuable information for the development of innovative therapeutic strategies. The HIF‐1α inhibitor LW6 effectively ameliorated silica dust‐induced pulmonary fibrosis in mice, either by interfering with silicosis progression or by promoting regression of established fibrosis. Additionally, our results show that HIF‐1α functions as a transcription factor, suppressing the mRNA expression levels of enzymes responsible for modifying the ECM. This suggests that HIF‐1α has a multifaceted role in fibrosis. Expanding on existing studies, this investigation provides a more in‐depth analysis of how HIF‐1α impacts ECM remodeling in pulmonary fibrosis, highlighting its potential as a therapeutic target.

**Figure 10:**
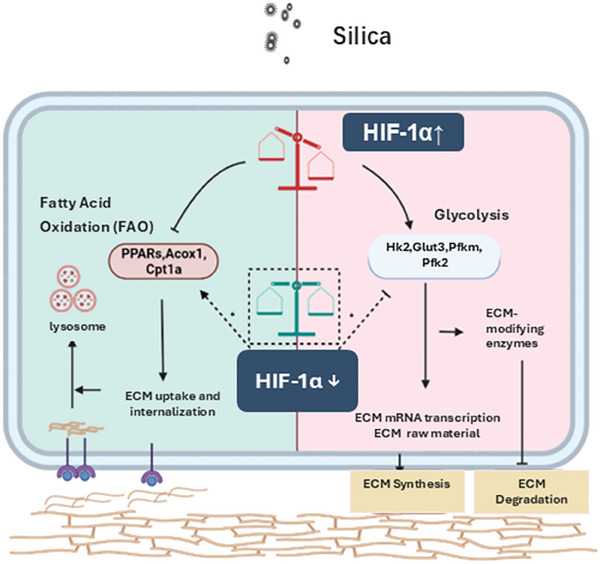
Schematic diagram of the mechanism of HIF‐1α regulating ECM deposition by regulating the glycolytic‐FAO metabolic pathway in silica‐induced pulmonary fibrosis.

Overall, our study reveals that increased glycolysis and decreased FAO contribute to the development of silica dust‐induced pulmonary fibrosis via promoting the dysregulation of ECM synthesis and degradation. Moreover, this study highlights the significance of HIF‐1α in ECM remodeling via regulating the FAO‐glycolysis metabolic shift (**Figure** **10**). Therefore, the utilization of metabolic therapy or the targeting of HIF‐1α presents a promising strategy for the eradication of fibrotic ECM to improve lung tissue functionality.

## 4 Experimental Section

### Animal Models

Male specific‐pathogen‐free (SPF) C57BL/6 mice were acquired from the Animal Core Facility of Nanjing Medical University (NJMU). All experiments in mice were approved by the Animal Ethical and Welfare Committee of NJMU (IACUC‐2204015) and were conducted following institutional procedures.

Experimental mice were induced lung fibrosis in accordance with our previous described steps.^[^ ^9^ ^]^ Experimental pulmonary fibrosis was induced in mice with 50 µL of 50 mg mL^−1^ silica suspended in sterile saline. 3PO (HY‐19824), pioglitazone (HY‐13956), and LW6 (HY‐13671), which were utilized in the mouse intervention and treatment studies, were procured from MedChemExpress (MCE).

### Cell Lines and Cell Transfection

MRC‐5 (human embryonic lung fibroblasts) cells were purchased from the Procell Life Science & Technology Co., Ltd. (China). MEM (Minimum Essential Medium) containing 10% fetal bovine serum and 1% penicillin‐streptomycin was used for MRC‐5 cell culture.

The siRNAs used in our study were designed and synthesized by GenePharma (China), and the riboFECT CP Transfection Kit (RiboBio) was used for cell transfection.

### RNA Extraction and Quantitative Real‐Time PCR (qRT‐PCR)

TRNzol universal reagent was used to isolate total RNA from cells and mouse lung tissues in accordance with the manufacturer's protocols. Briefly, cells and mouse lung tissues were harvested and homogenized in TRNzol universal reagent. Homogenized samples were left at room temperature for 5 min, and then added 0.2 mL of chloroform per 1 mL of TRNzol universal reagent. After being vigorously vortexed for 15 s, the samples were incubated at room temperature for 3 min. Centrifuged at 12 00 rpm at 4 °C for 15 min and removed the aqueous phase into clean tubes. Precipitated the RNA form the aqueous phase by mixing equal volume of isopropanol. After 10 min of incubation at room temperature, centrifuged at 12 00 rpm at 4 °C for 15 min. Removed the supernatant and washed the RNA pellet once with 75% ethanol. Dried the RNA pellet and added 20–30 µL RNase‐free water to dissolve RNA. RNA (500 ng) from cells or tissues was reversed‐transcribed using HiScript II Q Select RT SuperMix (Vazyme Biotech). The qRT‐PCR assay was conducted using SYBR Green 2×PCR mix (Vazyme Biotech) under a Roche LightCycler 480 II system.

### RNA‐Sequencing

Total RNA of mouse lung samples was extracted from control and silica‐inhaled mouse. RNA quality was evaluated by agarose gel electrophoresis and Nanodrop. RNA integrity numbers were assessed using Agilent 2100 Bioanalyzer. RNA‐seq libraries were generated using the NEBNext UltraTM RNA Library Prep Kit for Illumina following the manufacturer's instructions. Libraries were pooled and sequenced on an Illumina Novaseq 6000 platform.

After quality control of sequencing data, the clean reads were aligned to the mouse genome using Hisat2 (http://ccb.jhu.edu/software/hisat2). The Cufflinks software package (http://cole‐trapnell‐lab.github.io/cufflinks) was used for transcript assembly, quantification and differential expression analysis of RNA‐Seq. HTseq‐count (http://htseq.readthedocs.io/en/release_0.9.1) was used to quantify the number of reads mapping to the annotated genes. Fragments per Kilobase Million (FPKM) values were obtained through custom scripts.

### Untargeted Metabolomics Analysis

Snap‐frozen mouse lung tissues in liquid nitrogen were used to untargeted metabolomics detection. Metabolites extraction and LC‐MS/MS analysis were performed in cooperation with Shanghai BIOTREE Biological Technology Co., LTD (Shanghai, China).

### Protein Extraction and Western Blotting

For lung tissue protein extraction, T‐PER Tissue Protein Extraction Reagent (Thermo Scientific) was chosen to isolate the total protein. Radio immunoprecipitation assay (RIPA) buffer and phenylmethanesulfonyl fluoride (PMSF) (Beyotime) were used to extract total cell protein. For secreted protein, after the cells were cultured with serum‐free medium for 24 h, the serum‐free medium was collected and centrifuged. Added 1 volume of 100% Trichloroacetic acid (TCA) to 4 volumes of the serum‐free medium to precipitate secreted protein. Washed protein pellet with cold acetone 3 times before dissolved.

Western blot assays were performed to detect the relative protein expression. Briefly, denatured protein was separated via polyacrylamide gel electrophoresis and then transferred to polyvinylidene fluoride (PVDF) membrane. The membranes were blocked with 5% defatted milk and incubated with primary antibody at 4 °C overnight. Thereafter, the membranes were incubated with horseradish peroxidase‐labeled secondary antibodies. The antibodies as follow: Fibronectin (Abcam, Ab6328); Col1a1 (Abclonal, A1352); Elastin (ProteinTech, 15257‐1‐AP); HIF‐1α (Cell signaling Technology, 36 69); HK2 (ProteinTech, 22029‐1‐AP); GLUT3 (Abclonal, A4137); PFKM (Abclonal, A3617); PFK2 (Abclonal, A3934); ACOX1 (Abclonal, A21217); CPT1a (Abclonal, A5307); PPAR‐α (Abclonal, A3123); PPAR‐γ (Abclonal, A19676); GAPDH (Abclonal, AC033); β‐tubulin (HUABIO, ET1602‐4).

### ECM Protein Extraction

**Figure 11:**
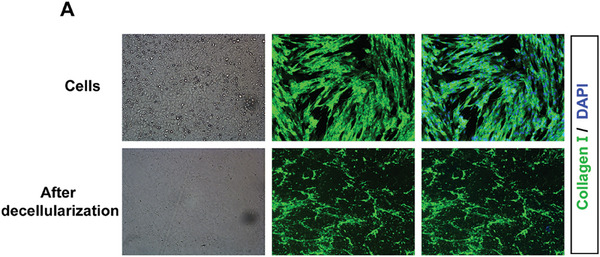
A) Representative images of cells with or without decellularization (left) and immunofluorescence staining for collagen I (green) in cells. Nuclei were stained with DAPI (blue).

For the extraction of fibroblast ECM proteins, we synthesized methodologies from previous studies and modified them to align with the specific characteristics of the cells under investigation.^[^ ^42^ ^]^ Fibroblasts were seeded in 35‐mm dishes until the cells grew to 90% confluency. Dishes were decellularized by incubation with 0.1% DOC (sodium deoxycholate) for 2 min, and then washed twice with phosphate buffered saline (PBS). Following the careful removal of PBS and subsequent microscopic examination, the fibrous structure was observed devoid of intact cells. Furthermore, the absence of DAPI fluorescence in the immunofluorescence staining post 4% formaldehyde fixation confirmed the successful decellularization and isolation of the ECM proteins. For western blot analysis, sample buffer fited with 1 mM dithiothreitol (DTT) was added, ECM was scraped off, and proteins were separated by sodium dodecyl sulfate polyacrylamide gel electrophoresis‌ (SDS‐PAGE) followed by immunoblotting.(**Figure** **11**)

### Histopathology

Fresh mouse lung tissues were fixed in 4% paraformaldehyde and embedded in paraffin. Hematoxylin and eosin (H&E) staining, Masson staining, Sirius red staining and Immunohistochemical staining were performed in cooperation with Wuhan Servicebio Biotechnology Co., LTD according to standard operating procedures. The lungs' pathological changes were measured according to the degree of alveolar wall thickening, cellular proliferation, inflammatory lesions, the deposition of collagen, and the extent of fibrotic lesions via H&E staining. The classification criteria are as follows: The severity of lesion: 0 = nothing/zero, 1 = marginal, 2 = slight, 3 = moderate, 4 = severe, and 5 = very severe. The distribution of lesion: 0 = absent, 1 = rare/occasional (10% of the lung area), 2 = sparse/ limited (10%–25% of the lung area), 3 = moderate (25%–50% of the lung area), 4 = extensive/widespread (50%–75% of the lung area), and 5 = very extensive/predominant (over 75% of the lung area).

### Soluble Collagen Assay

The quantity of soluble collagen present in the culture medium was evaluated utilizing the Sircol Soluble Collagen Assay (Biocolor) in accordance with the guidelines provided by the manufacturer.

### Cellular Metabolism Assays

Glycolysis and FAO functional assessment for lung fibroblast were detected on an XFe96 analyzer (Agilent) following the manufacturer's protocol. Cells were cultured on an XF96 plate. For detection of glycolysis, a final concentration of 10 mM glucose, 1 µM oligomycin, and 100 mM 2‐Deoxy‐Glucose (2‐DG) was used. For FAO measurement, Etomoxir (4 µM), oligomycin (2 µM), carbonyl cyanide‐p‐trifluoromethoxyphenylhydrazone (FCCP) (1 µM), antimycin A (0.5 µM) and rotenone (0.5 µM) were used.

### Mitochondrial and Lysosomal Staining

Cells were plated on confocal dishes (Corning) and treated according to experimental requirements. For quantification of mitochondria, cells were exposed to Mito‐Tracker (Beyotime) for 20 min as per protocol. After the cells were fixed by 4% paraformaldehyde, actin protein was stained using Actin‐Tacker (Beyotime) in accordance with manufacturer's instructions. For lysosome quantification, cells were incubated with Lyso‐Tracker (Beyotime) following protocol and then fixed to further staining. Images was captured using a Zeiss laser‐scanning confocal microscope.

### Glycine Assay and LOX Enzyme Activity Assay

The concentration of glycine (GLY) was detected using a Glycine assay kit (ab211100, Abcam) as per the standard procedures. Lysyl oxidase activity in cells and culture medium was determined by LOX Activity Assay Kit (Fluorometric, Abcam, ab112139). According to the manufacturer's plan, the activity of LOX enzyme is detected using a red fluorescence substrate for HRP‐coupled reactions at Ex/Em = 540/590 nm in a fluorescence microplate reader.

### Immunofluorescent Staining

For immunofluorescent staining of cells, cells were fixed using 4% paraformaldehyde and then blocked with 10% goat serum. Next, cells were incubated with primary antibody and subsequently specifically bound to FITC or CY3‐conjugated secondary antibody (Beyotime). Nucleus was stained suing DAPI (Beyotime). After adding a drop of Antifade Mounting Medium (Beyotime), cells were observed under a fluorescence microscope (Nikon).

### Collagen Hybridizing Peptide (CHP) Staining

Cy3‐conjugated CHP reagents were acquired from 3Helix Inc. Fixed mouse lung tissues were soaked in 30% sucrose solution until tissues sank to the bottom of the tube. After embedding with OCT (optimal cutting temperature compound), tissues were sectioned into 10 µm‐thick slices. Removed the embedding OCT compound via washing 3 times with PBS. Since CHP was low non‐specific binding to tissue, blocking with 10% goat serum can be omitted. According to the instructions, CHP tended to slowly self‐assemble into CHP triple helices in solution during storage. Therefore, the trimeric CHP must be dissociated to monomers by heating prior to use. Heated the dilute CHP solution (10 µM) at 80 °C for 5 min and immediately immerse the CHP microtube in an ice‐water bath to quench the solution to room temperature. Subsequently pipetted the solution onto each lung tissue section quickly and incubated the tissues with the staining solution at 4 °C overnight. After staining, washed the lung tissue slides with PBS 3 times and counterstained with DAPI. Images were captured using a Zeiss laser‐scanning confocal microscope.

### Statistical Analysis

All the data were presented by means ± SD, and all experiments were repeated at least 3 times. The independent‐samples t‐test was used to analyze 2 groups, and one‐way analysis of variance (ANOVA) was used to analyze more groups with Dunnett's test. *p* < 0.05 was considered significant.

### Ethics Approval and Consent to Participate

All in vivo experiments were conducted following the agreements authorized by the Laboratory Animal Welfare Ethics Committee of Nanjing Medical University (IACUC‐2204015).

## Conflict of Interest

The authors declare no conflict of interest.

## Supporting information



Supporting Information

## Data Availability

The data that support the findings of this study are available from the corresponding author upon reasonable request.
