# Supplementary material for: Fatty Acid Oxidation‐Glycolysis Metabolic Transition Affects ECM Homeostasis in Silica‐Induced Pulmonary Fibrosis
Source: Adv Sci (Weinh). 2024 Dec 25;12(7):2407134. doi: 10.1002/advs.202407134 (PMC11831484; doi:10.1002/advs.202407134)
Supplement: Supplementary file 1 — Supporting Information [file ADVS-12-2407134-s001.docx]

**Fatty acid oxidation-glycolysis metabolic transition affects ECM homeostasis in silica-induced pulmonary fibrosis.**

*Wenqing Sun^1, #^; Siyun Zhou ^2, #^; Lan Peng^2, #^; Wei Wang^3, #^; Yi Liu^2^; Ting Wang^4^; Demin Cheng^2^; Ziwei Li^2^; Haojie Xiong^2^; Xinyin Jia^2^; Wenxiu Lian^2^; Jiandong Jiao^3, *^; Chunhui Ni^5, *^*

^1^The Affiliated Wuxi Center for Disease Control and Prevention of Nanjing Medical University, Wuxi Center for Disease Control and Prevention, Wuxi Medical Center, Nanjing medical university, Wuxi, China.

^2^Department of Occupational Medical and Environmental Health, Key Laboratory of Modern Toxicology of Ministry of Education, Center for Global Health, School of Public Health, Nanjing Medical University, Nanjing, 211166, China

^3^The Affiliated Wuxi Center for Disease Control and Prevention of Nanjing Medical University, Wuxi Center for Disease Control and Prevention, Wuxi Medical Center, Nanjing Medical University.

^4^Department of Occupational Medical and Environmental Health, Key Laboratory of Modern Toxicology of Ministry of Education, Center for Global Health, School of Public Health, Nanjing Medical University, Nanjing 211166, China; Department of Pathology, Nanjing Drum Tower Hospital, The Affiliated Hospital of Nanjing University Medical School, Nanjing 210000, China.

^5^Department of Occupational Medical and Environmental Health, Key Laboratory of Modern Toxicology of Ministry of Education, Center for Global Health, School of Public Health, Nanjing Medical University, Nanjing 211166, China; Department of Public Health, Kangda College of Nanjing Medical University, Lianyungang 320700, China. Electronic address: [chni@njmu.edu.cn](mailto:chni@njmu.edu.cn).

^#^These authors contributed equally to this work and should be considered co-first authors.

^*^These authors should be regarded as co-corresponding authors. Correspondence to: Professor Chunhui Ni, 818 Tianyuan East Road, Nanjing 211166, China. Email: [chni@njmu.edu.cn](mailto:chni@njmu.edu.cn), [chni@njmu.edu.cn](mailto:chni@njmu.edu.cn). Professor Jiandong Jiao, 499 Jincheng Road, Wuxi 214023, China. Email: jiaojiandong@njmu.edu.cn.

**Figures S1-7 Legends**

**Figure S1:** The development of pulmonary fibrosis in mice is accompanied by a restructuring of cellular metabolism.

**Figure S2:** The deposition of ECM by activated fibroblasts is accomplished by a metabolic perturbation of glycolysis and FAO.

**Figure S3:** Inhibiting glycolysis or enhancing FAO affects ECM deposition in activated fibroblasts.

**Figure S4:** Cellular metabolism assays after inhibiting glycolysis or enhancing FAO.

**Figure S5:** HIF-1α regulated the transformation of FAO-glycolysis in fibroblast.

**Figure S6:** HIF-1α inhibition reduced ECM deposition by regulating glycolytic-FAO metabolic disturbance.

**Figure S7:** Safety assessment of 3PO plus pioglitazone and LW6 treatments in the pulmonary fibrosis mouse model.


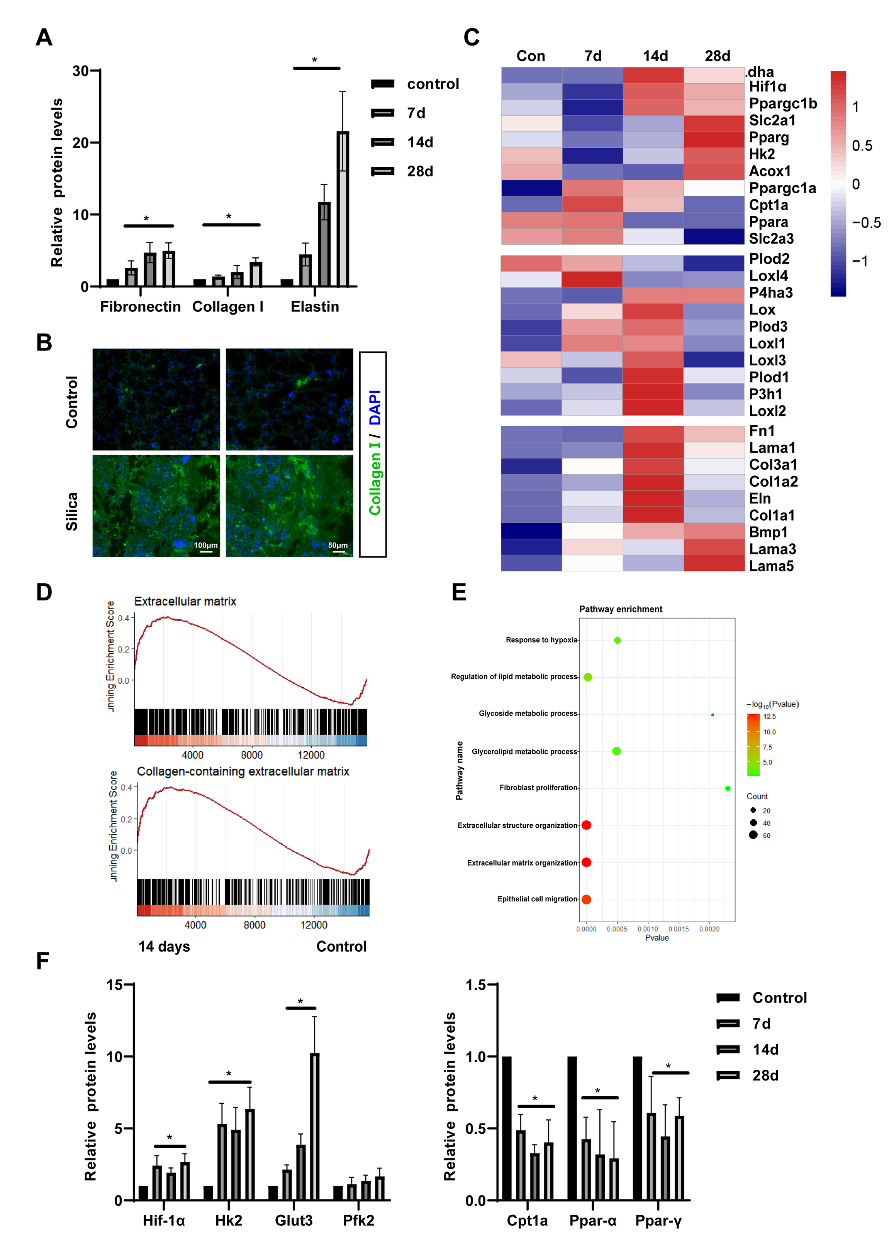
Figure S1

(A) Quantification of immunoblots in Fig. 1C

(B) Representative image showing collagen Ⅰ (green) stained with anti- collagen Ⅰ antibody in mouse lung tissue. Nuclei were stained with DAPI (blue).

(C) The heatmap of differential expression of fibrotic genes, ECM related genes and key molecules involved in glycolysis and fatty acid oxidation identified by transcriptomics. Heatmap scale from red to blue where red indicates higher expression, and blue reflects lower expression.

(D) GSEA analysis of extracellular matrix and collagen-containing extracellular matrix.

(E) GO pathway enrichment analysis.

(F) Quantification of immunoblots in Fig. 1F


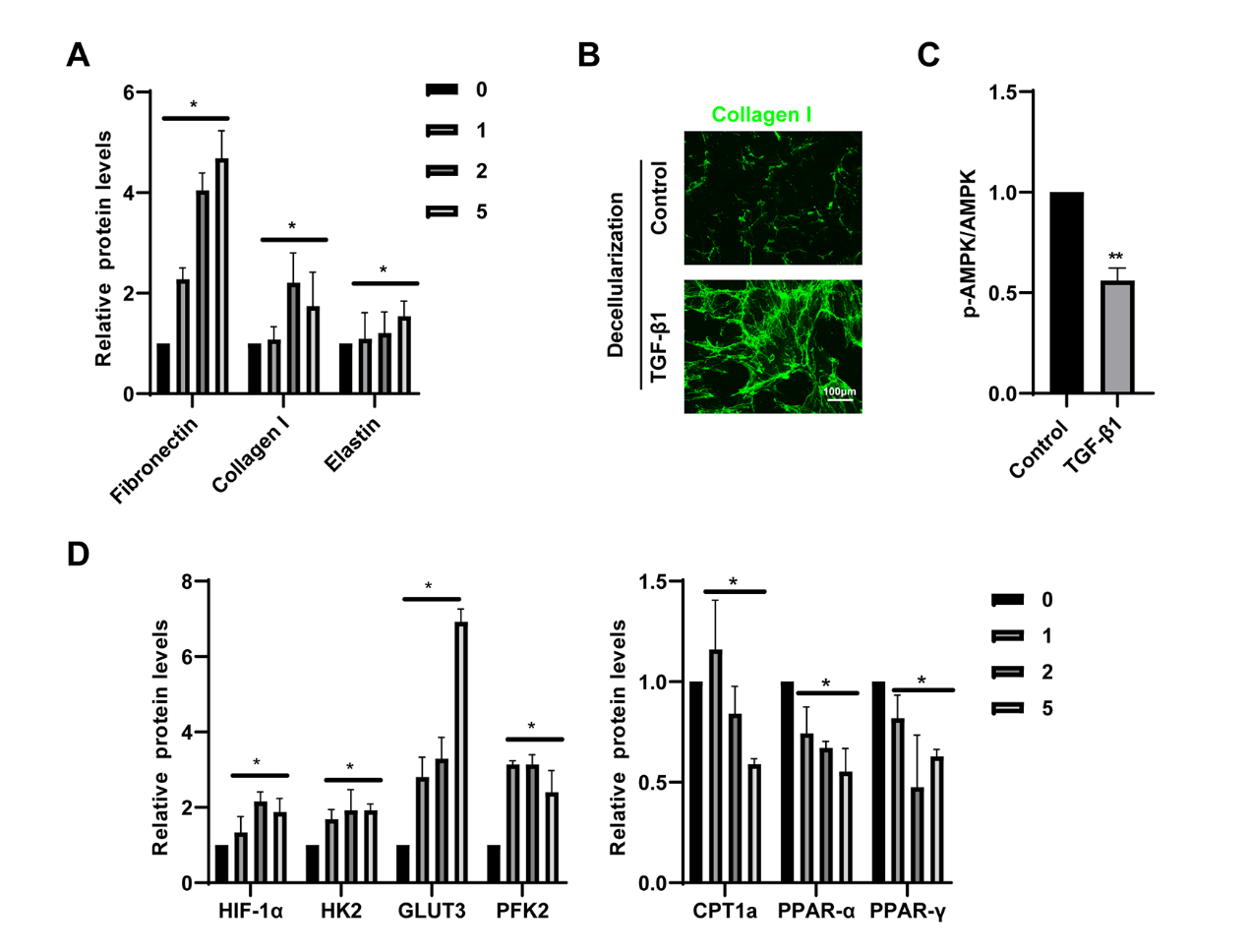


Figure S2

(A) Relative expression of core components of ECM in MRC-5 cells treated with different TGF-β1 concentration by qRT-PCR. All data were expressed as the means ± SD of at least 3 independent experiments, **p* < 0.05 and ***p* < 0.01.

(B) Immunofluorescence images showing fluorescence intensity of Collagen I after decellularization.

(C) Quantification of immunoblots in Fig. 2E

(D) Quantification of immunoblots in Fig. 2G


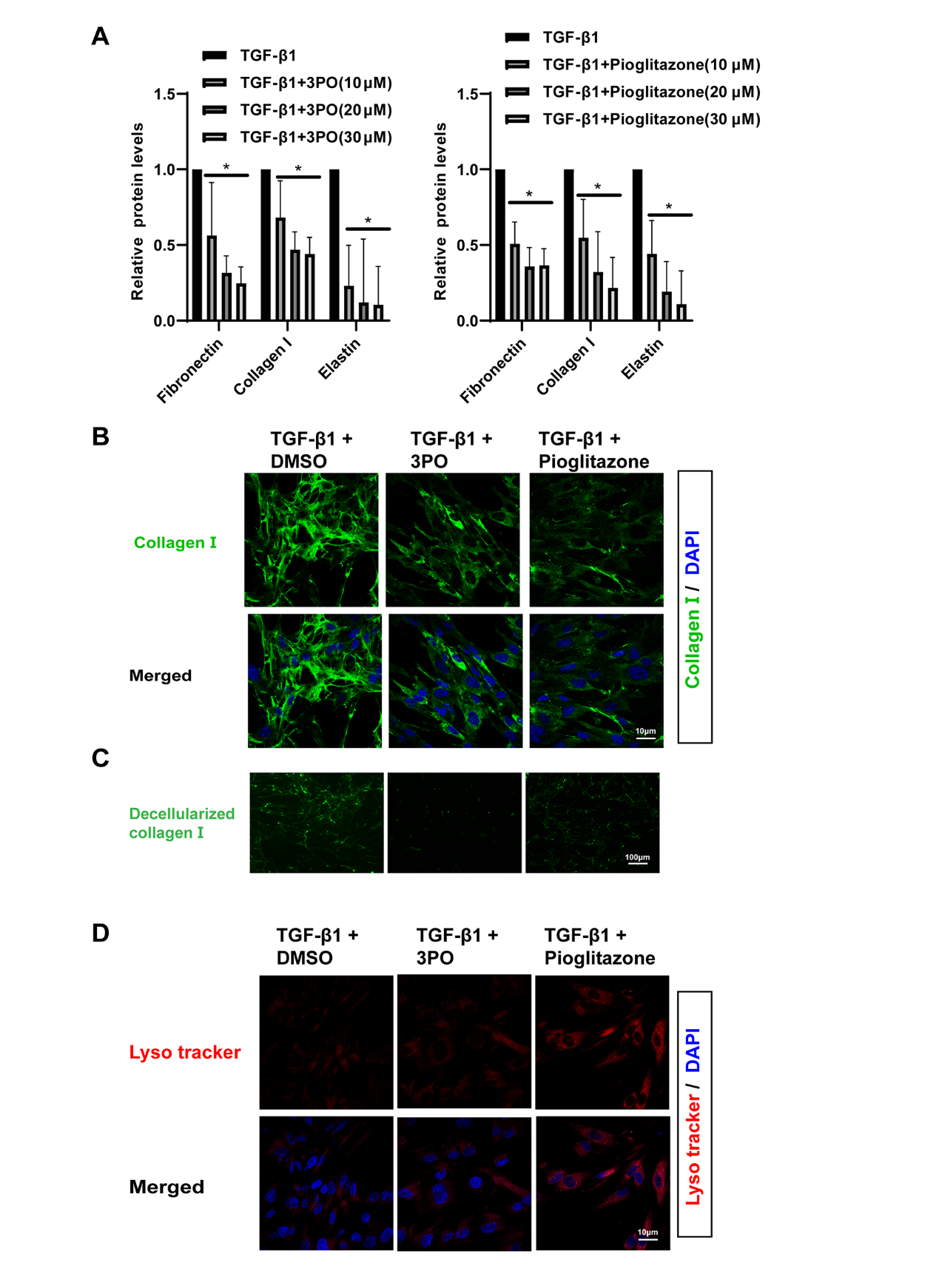


Figure S3

(A) Quantification of immunoblots in Fig. 3A

(B) Representative image showing collagen Ⅰ (green) stained with anti- collagen Ⅰ antibody in MRC-5 cells after TGF-β1 treatment. Nuclei were stained with DAPI (blue).

(C) Immunofluorescence images showing fluorescence intensity of Collagen I after decellularization.

(D) Confocal fluorescence images of MRC-5 cells incubated with lyso-Tracker.


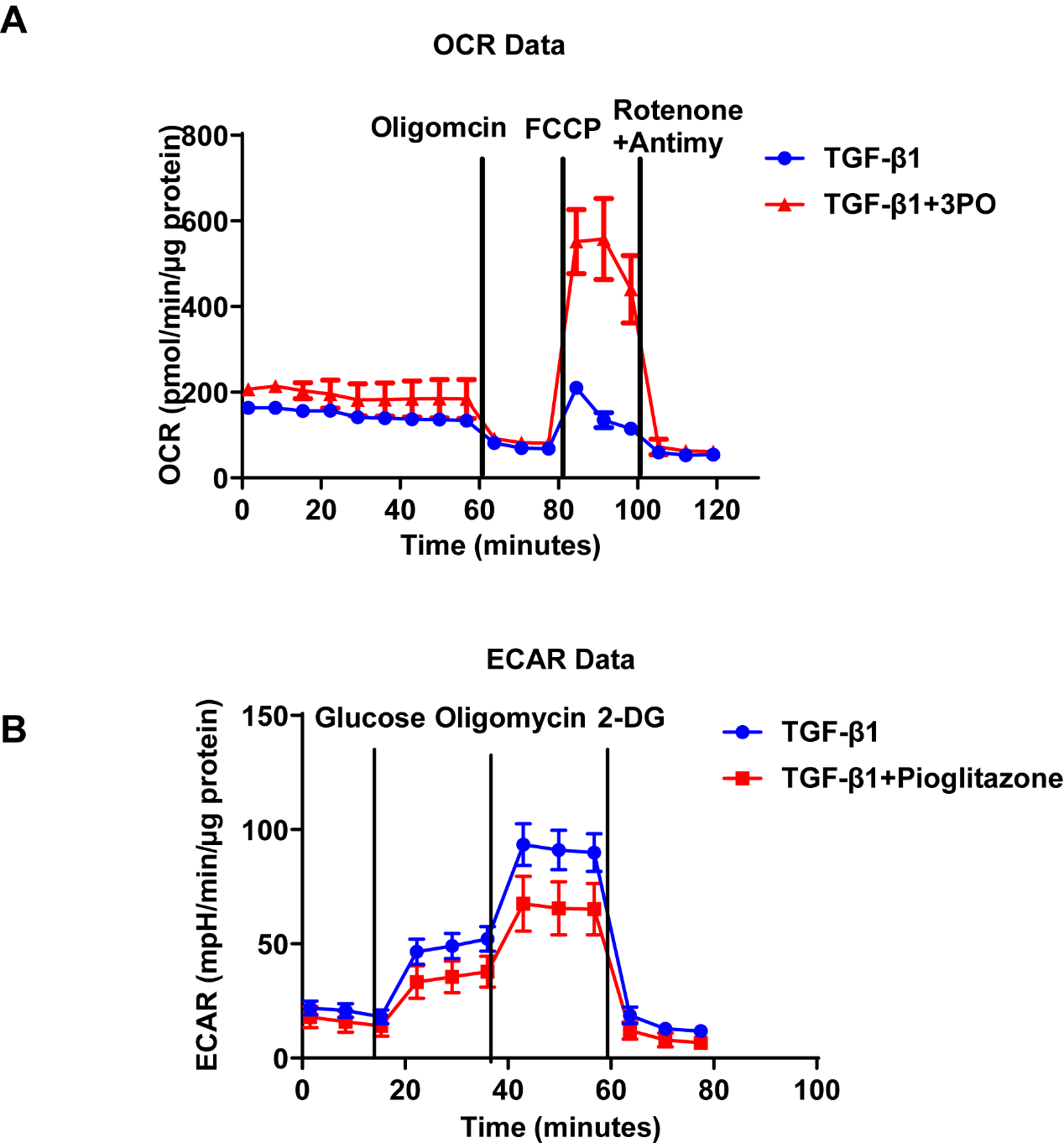


Figure S4

(A) Real-time measurements to determine the effect of 3PO treatment on OCR in MRC-5 cells.

(B) Real-time measurements to determine the effect of pioglitazone treatment on ECAR in MRC-5 cells.


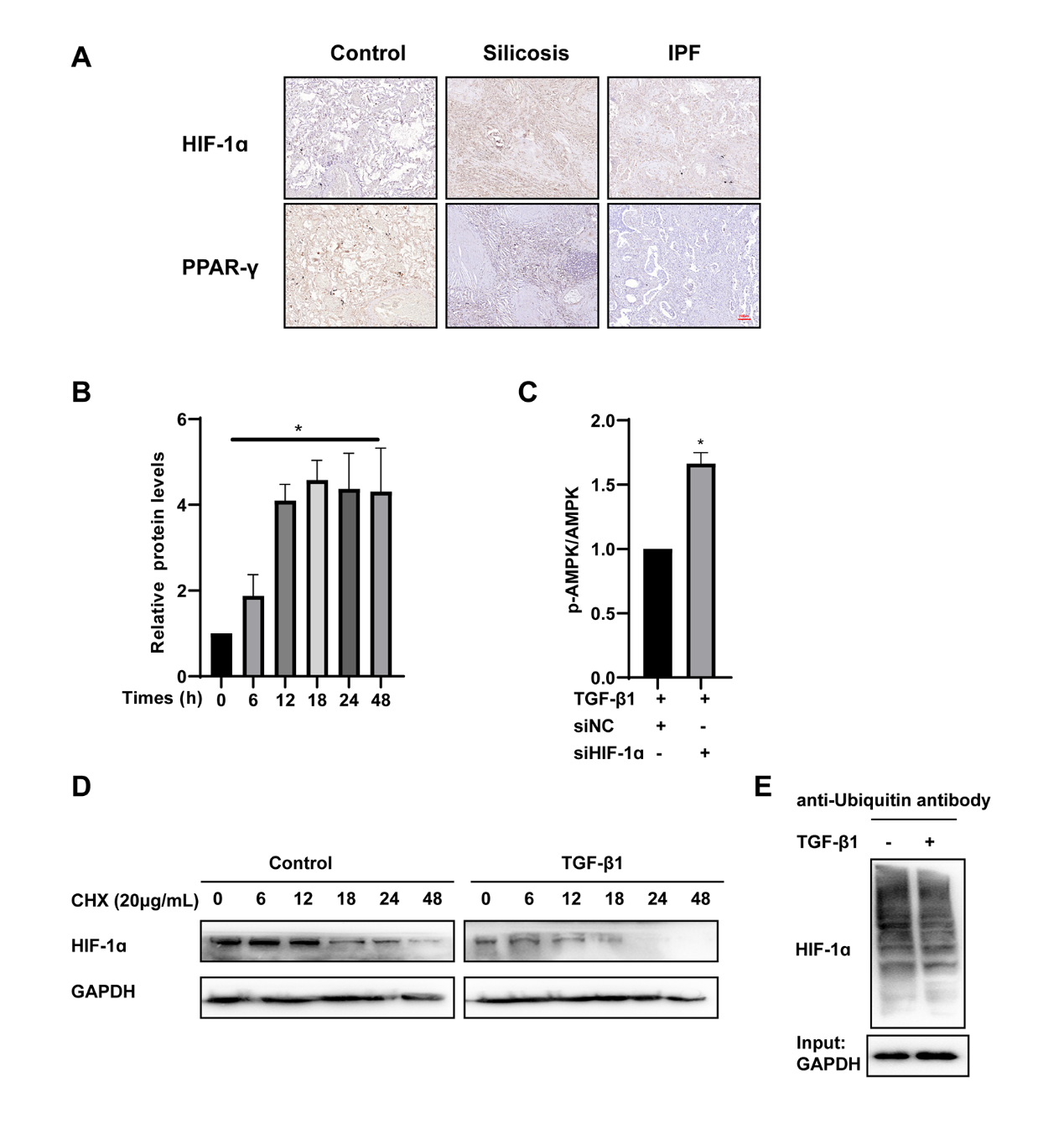


Figure S5.

(A) Immunohistochemical staining for Hif-1α and PPAR-γ in lung tissue sections from silicosis and IPF patients,

(B) Quantification of immunoblots in Figure 6C.

(C) Quantification of immunoblots in Figure 6F.

(D) The protein levels of HIF-1α in MRC-5 cells at different time-points after 20 μg/mL CHX treatment were examined by the western blot. The results of the experiment were repeated at least three times.

(E) Western blot analysis for detecting the ubiquitination level of HIF-1α.


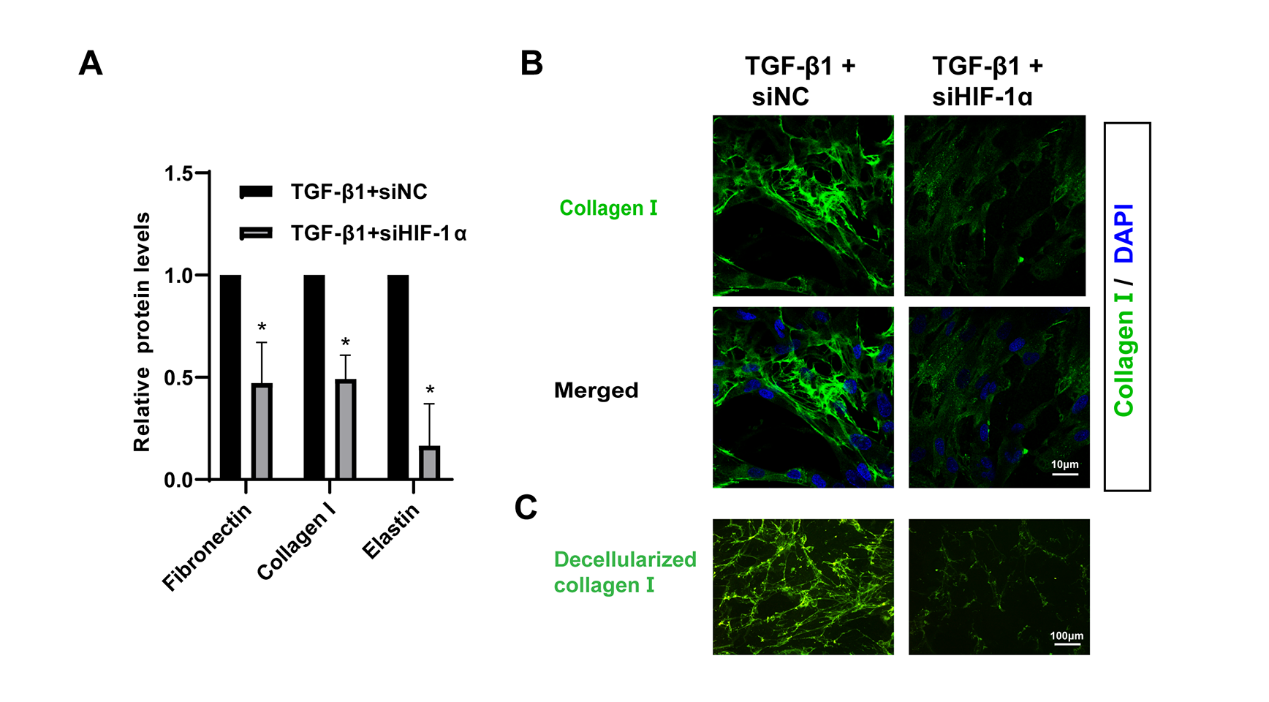
Figure S6.

(A) Quantification of immunoblots in Figure 7A.

(B) Representative image showing collagen Ⅰ (green) stained with anti- collagen Ⅰ antibody in MRC-5 cells after TGF-β1 and siRNA treatment. Nuclei were stained with DAPI (blue).

(C) Immunofluorescence images showing fluorescence intensity of Collagen I after decellularization.


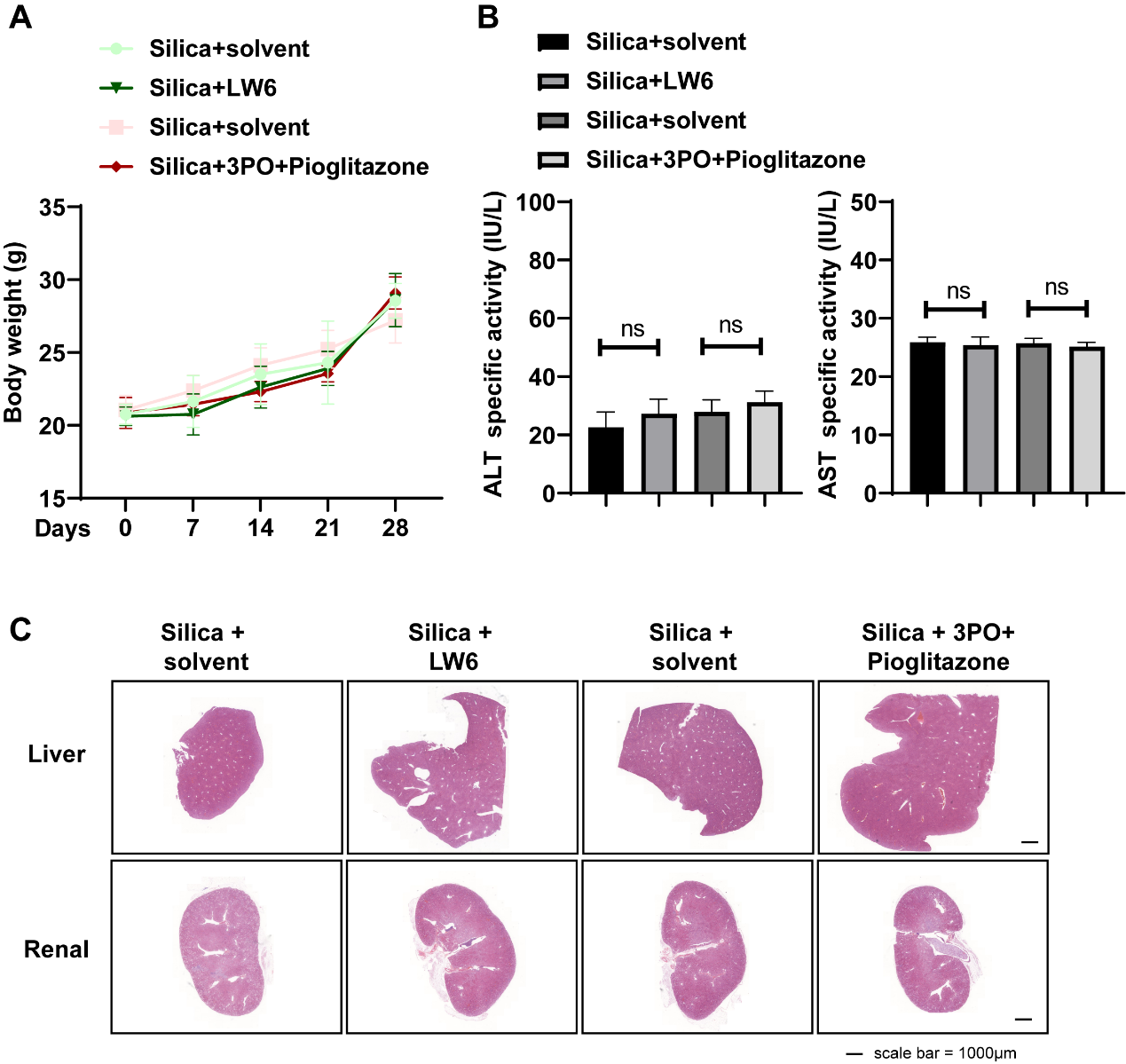


Figure S7.

(A) Body weight changes of mice.

(B) Levels of mouse serum ALT (left) and AST (right).

(C) Histological analysis was conducted on mouse liver and renal tissues using H&E staining.
